# Inhalable ROS‐Responsive Nanospray Activates PPAR‐γ to Restore Macrophage Mitochondrial Homeostasis and Attenuate Radiation‐Induced Lung Injury

**DOI:** 10.1002/advs.76447

**Published:** 2026-07-08

**Authors:** Mingquan Gao, Xudong Yu, Ziqian Shang, Mengyao Tan, Xie Huang, Zaizhi Du, Xiaojiao Wang, Xinrui Yang, Ximei Luo, Weidong Wang, Rong Li, Shenglin Luo

**Affiliations:** ^1^ Department of Military Preventive Medicine Institute of Combined Injury State Key Laboratory of Trauma and Chemical Poisoning Chongqing Engineering Research Center for Nanomedicine Third Military Medical University (Army Medical University) Chongqing China; ^2^ Institute of Fundamental and Frontier Sciences University of Electronic Science and Technology of China Chengdu China; ^3^ Department of Radiation Oncology Radiation Oncology Key Laboratory of Sichuan Province Sichuan Clinical Research Center for Cancer Sichuan Cancer Hospital & Institute Affiliated Cancer Hospital of University of Electronic Science and Technology of China Chengdu China

**Keywords:** astaxanthin, macrophage reprogramming, mitochondrial quality control, NAD^+^, PPAR‐γ activation, radiation‐induced lung injury

## Abstract

Radiation‐induced lung injury (RILI) is a major complication of nuclear radiation exposure and thoracic radiotherapy, driven in part by a vicious cycle of oxidative stress and mitochondrial dysfunction. Peroxisome proliferator‐activated receptor gamma (PPAR‐γ), a key regulator of mitochondrial homeostasis and inflammatory resolution, therefore represents a potential therapeutic target, yet multi‐omics analyses revealed that this immunometabolic checkpoint remains functionally constrained in irradiated macrophages. To reactivate this pathway, we developed an inhalable, ROS‐responsive nanospray (HANP) by loading nicotinamide adenine dinucleotide (NAD^+^) and astaxanthin (ASX) into a hollow mesoporous polydopamine (HMPDA) shell. Excess ROS in microenvironment triggers oxidative degradation of the HMPDA shell, enabling intracellular release of NAD^+^ and ASX. Mechanistically, NAD^+^ promotes SIRT1‐mediated deacetylation of PPAR‐γ, whereas ASX serves as an activating ligand, thereby cooperatively restoring PPAR‐γ signaling. This response re‐establishes macrophage mitochondrial homeostasis by coordinating mitochondrial biogenesis and mitophagy, which subsequently promotes a shift toward a reparative macrophage phenotype. In murine models of focal thoracic irradiation and lethal whole‐body irradiation, HANP markedly attenuated lung injury and improved survival. Collectively, these findings identify macrophage PPAR‐γ as a therapeutically actionable redox‐immunometabolic regulator and support inhaled dual‐activation of this pathway as a promising strategy for protection against RILI in both clinical and emergency settings.

## Graphical abstract

A nebulized nanoassembly alleviates radiation‐induced lung injury by reprogramming macrophages through coordinated ROS scavenging, NAD^+^ replenishment, and PPAR‐γ activation. Redox balance, mitochondrial homeostasis, and immune regulation are restored, revealing a microenvironment‐centered strategy for localized radioprotection.

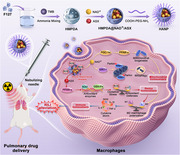

## 1 Introduction

The escalating threat of nuclear accidents, radiological terrorism, and the strategic necessities of military defense, combined with the widespread application of thoracic radiotherapy in oncology, highlight the urgency of developing effective medical countermeasures against radiation‐induced lung injury (RILI) [1]. As a dose‐limiting toxicity, RILI is not merely a transient inflammatory response but a complex, progressive pathology initiated by high‐energy ionizing radiation (IR) [2]. The deposition of energy rapidly induces DNA double‐strand breaks and generates a surge of reactive oxygen species (ROS), triggering a cascade of molecular events that culminate in acute pneumonitis and chronic pulmonary fibrosis [3]. Despite advances in precision radiotherapy, the incidence of symptomatic RILI remains high, significantly compromising patients’ quality of life and survival [4]. Currently, clinical management relies heavily on corticosteroids or the radioprotectant amifostine [5]. However, these interventions are severely limited by narrow therapeutic windows, non‐specific immune suppression, and systemic toxicity [6]. Furthermore, they often fail to arrest the self‐perpetuating cycle of oxidative stress and metabolic collapse that drives the chronic phase of the injury [7]. Consequently, there is a critical unmet need to decipher the molecular checkpoints governing radiation resistance and to engineer targeted strategies that can reactivate endogenous defense mechanisms in both emergency and clinical settings.

Macrophages, the most abundant immune cells in the lung, serve as the central orchestrators of pulmonary homeostasis and injury repair [8, 9, 10]. Their functional plasticity allows them to transition from a pro‐inflammatory M1 phenotype, which is essential for early pathogen defense but destructive if persistent, to a reparative M2 phenotype that promotes tissue resolution [11, 12, 13]. Recent advances in immunometabolism have revealed that this phenotypic switch is intrinsically dictated by mitochondrial quality control—a delicate balance between mitochondrial biogenesis and the clearance of damaged organelles via mitophagy [14, 15]. In the context of RILI, IR‐induced oxidative stress disrupts the electron transport chain, causing mitochondrial depolarization and ATP depletion [16]. This metabolic catastrophe locks macrophages in a glycolytic, hyper‐inflammatory M1 state, preventing the metabolic shift toward oxidative phosphorylation required for M2 polarization [17, 18]. Therefore, restoring macrophage mitochondrial homeostasis represents a promising, yet underexplored, therapeutic avenue to break the cycle of radiation injury.

To identify specific molecular targets capable of reversing this metabolic impairment, we integrated single‐cell RNA sequencing (scRNA‐seq) and bulk transcriptomics analyses of irradiated lung tissues. Our integrative transcriptomic analysis approach pinpointed peroxisome proliferator‐activated receptor‐gamma (PPAR‐γ) as a unique metabolic checkpoint. Unlike classical inflammatory mediators that are drastically upregulated, PPAR‐γ expression remains stable but functionally “dormant” within pulmonary macrophages under radiation stress. As a master regulator of lipid metabolism and mitochondrial biogenesis, PPAR‐γ theoretically possesses the capacity to resolve inflammation and restore energy balance [19, 20]. Although synthetic PPAR‐γ agonists (e.g., rosiglitazone and pioglitazone) have shown potential in mitigating radiation injury in other organs, their clinical translation for RILI is severely hindered [21, 22]. Conventional systemic administration often leads to insufficient pulmonary accumulation while triggering severe off‐target effects, including weight gain, cardiovascular complications, and skeletal fragility [23, 24]. Furthermore, the harsh oxidative microenvironment in RILI lesions directly oxidizes PPAR‐γ and depletes its essential cofactor, nicotinamide adenine dinucleotide (NAD^+^), rendering endogenous or systemic activation ineffective [25, 26, 27]. Thus, a strategy that simply provides an agonist via conventional routes is insufficient; effective therapy requires a “multi‐pronged” approach that simultaneously remodels the oxidative microenvironment, replenishes metabolic cofactors, and precisely delivers the activator to pulmonary macrophages via non‐invasive targeted routes.

**SCHEME 1:**
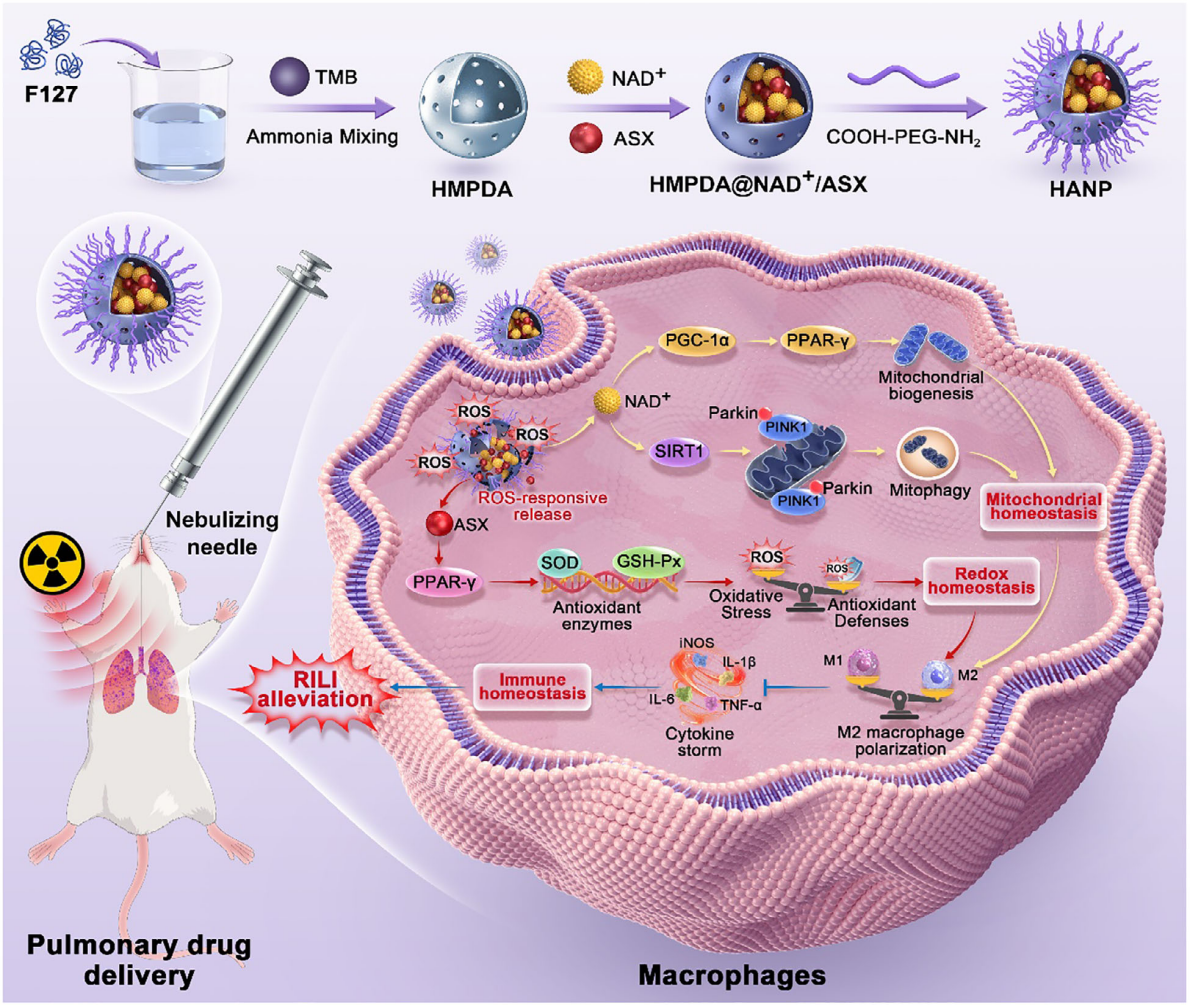
Schematic illustration of the ROS‐responsive HANP nanospray for RILI alleviation. HANP is synthesized by co‐loading NAD^+^ and ASX into PEGylated HMPDA. Upon nebulization, the HMPDA shell scavenges microenvironmental ROS and undergoes oxidative degradation to trigger payload release within the lungs. Inside macrophages, these agents synergistically reactivate the dormant PPAR‐γ axis, restoring mitochondrial homeostasis (through biogenesis and mitophagy) and redox balance. This metabolic shift drives macrophage polarization from a pro‐inflammatory M1 to a reparative M2 phenotype, effectively suppressing the cytokine storm to mitigate RILI.

To break this pathological cycle and specifically activate the dormant PPAR‐γ receptor, we developed an inhalable, ROS‐responsive nanospray (HANP). This system utilizes a hollow mesoporous polydopamine (HMPDA) shell to co‐deliver NAD^+^ and astaxanthin (ASX) directly to the alveolar microenvironment. Upon deposition in the inflamed lungs, the HMPDA shell scavenges the abundant local ROS. Importantly, this oxidation process directly triggers the degradation of the shell, leading to the on‐demand, intracellular release of the encapsulated payloads. Once released, NAD^+^ and ASX work in tandem to rescue the compromised SIRT1–PPAR‐γ axis. Specifically, the released NAD^+^ replenishes the depleted cellular pool to fuel SIRT1 (an NAD^+^‐dependent deacetylase), enabling it to deacetylate PPAR‐γ. Simultaneously, ASX binds to this deacetylated receptor as a direct activating ligand. This dual‐activation synchronizes mitochondrial biogenesis and mitophagy, successfully steering macrophages toward a reparative phenotype (Scheme 1). Ultimately, HANP demonstrated robust radioprotection in murine models, offering a highly translatable, non‐invasive intervention for clinical RILI management.

## 2 Results

### 2.1 Irradiation Preserves PPAR‐γ Expression but Suppresses Its Transcriptional Activity and Downstream Signaling

**FIGURE 1:**
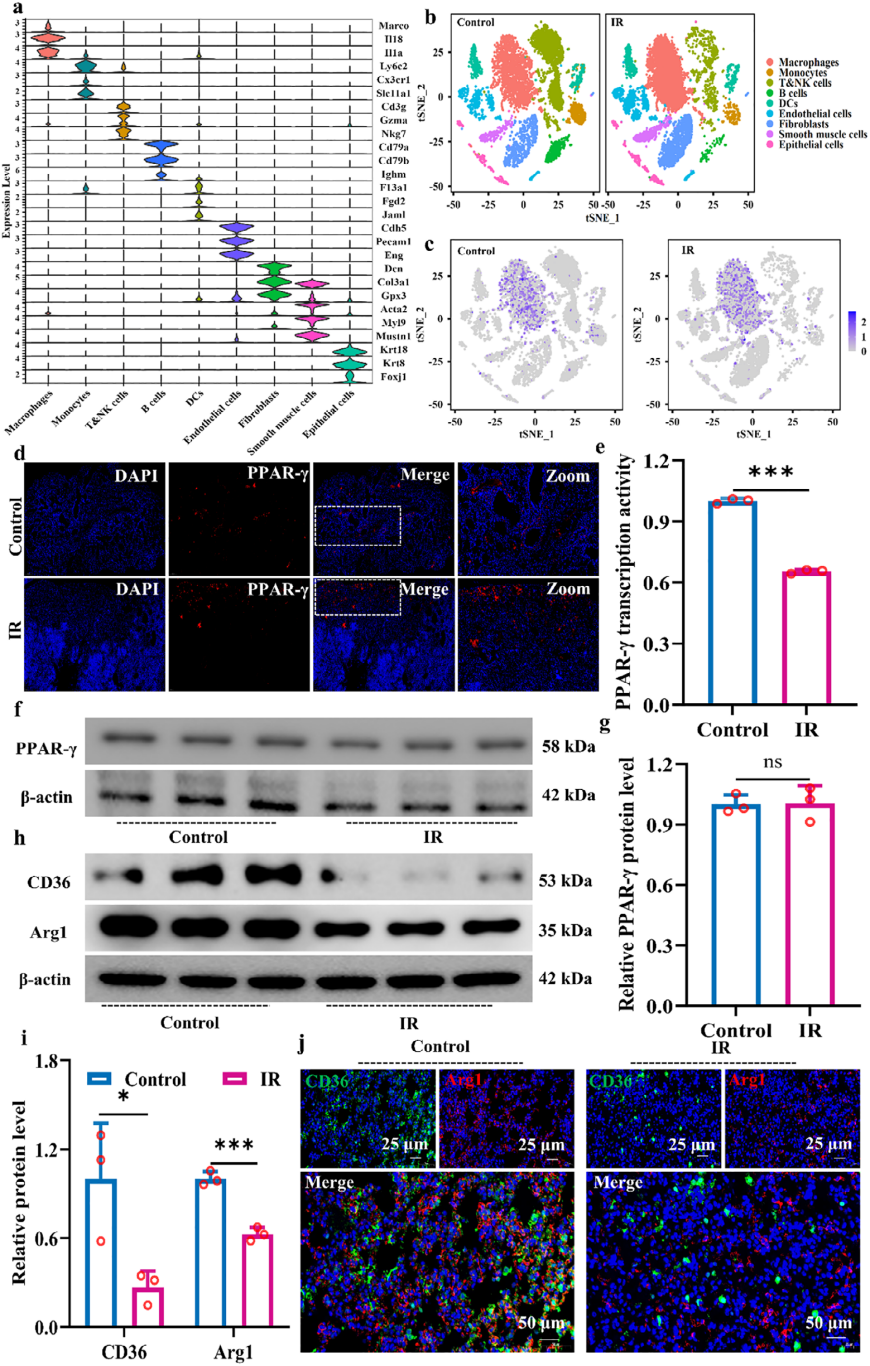
Irradiation disrupts PPAR‐γ functional output without markedly altering its expression. (a) Violin plot showing the expression of representative marker genes across major lung cell populations identified by single‐cell RNA‐seq analysis. (b) t‐SNE plots showing the cellular composition of control and irradiated lungs. (c) Feature plots showing the distribution of PPAR‐γ expression in control and irradiated lungs. (d) Representative immunofluorescence images of PPAR‐γ in lung tissues from control and irradiated mice. Enlarged views of the boxed regions are shown in the zoom panels. (e) Quantification of PPAR‐γ transcription factor activity in control and irradiated MH‐S cells. (f–i) Representative Western blot images and quantitative analysis of PPAR‐γ and its downstream target proteins CD36 and Arg1 in control and irradiated MH‐S cells. (j) Representative immunofluorescence staining of CD36 and Arg1 in lung tissues from control and irradiated mice. Scale bars: 25 and 50 µm as indicated. Data are presented as mean ± s.d. Statistical analysis was performed using independent samples *t*‐test. *p < 0.05, **p < 0.01, ***p < 0.001.

To identify therapeutically relevant molecular candidates in RILI, three independent lung transcriptomic datasets (GSE85359, GSE41789, and GSE25295) were integrated and analyzed. Following batch‐effect correction (Figure S1a), differential expression analysis revealed that while IR induced broad transcriptional changes, *Pparg* (encoding PPAR‐γ) expression remained largely unchanged between control and IR groups (Figure S1b,c). To resolve the cellular atlas of PPAR‐γ, single‐cell RNA‐seq analysis (GSE206426) was performed, identifying nine distinct pulmonary cell populations (Figure 1a,b and Figure S1d). Notably, *Pparg* was most prominently expressed in macrophages, with its transcriptional levels remaining minimally changed after IR across all identified cell types (Figure 1c and Figure S1e). This stability was further confirmed at the protein level; Western blot and immunofluorescence assays demonstrated that IR did not significantly alter PPAR‐γ abundance, spatial distribution, or expression intensity in lung tissues (Figure 1d–g).

To determine whether irradiation affects PPAR γ expression or functional activity in macrophages, we further examined PPAR γ status in MH‐S cells. Immunofluorescence analysis showed that irradiation did not obviously alter PPAR γ expression or nuclear localization (Figure S2a). In contrast, transcription factor activity assay revealed a significant reduction in PPAR γ activity after irradiation (Figure 1e). Consistently, although total PPAR γ protein levels remained largely unchanged, acetylated PPAR γ was markedly increased in irradiated cells (Figure S2b,c). Moreover, the expression of the PPAR γ downstream target genes CD36 and Arg1 was significantly reduced in irradiated MH‐S cells, and a similar decrease was also observed in lung tissue (Figure 1h–j). These data indicate that irradiation impairs PPAR γ function primarily at the activity level rather than by reducing its total protein abundance. Given the established role of PPAR‐γ in restraining inflammation and oxidative stress, we therefore hypothesized that enhancing PPAR‐γ activity, rather than increasing its expression, may represent a rational strategy to mitigate RILI progression.

### 2.2 Synthesis and Physicochemical Characterization of ROS‐Responsive HANP

**FIGURE 2:**
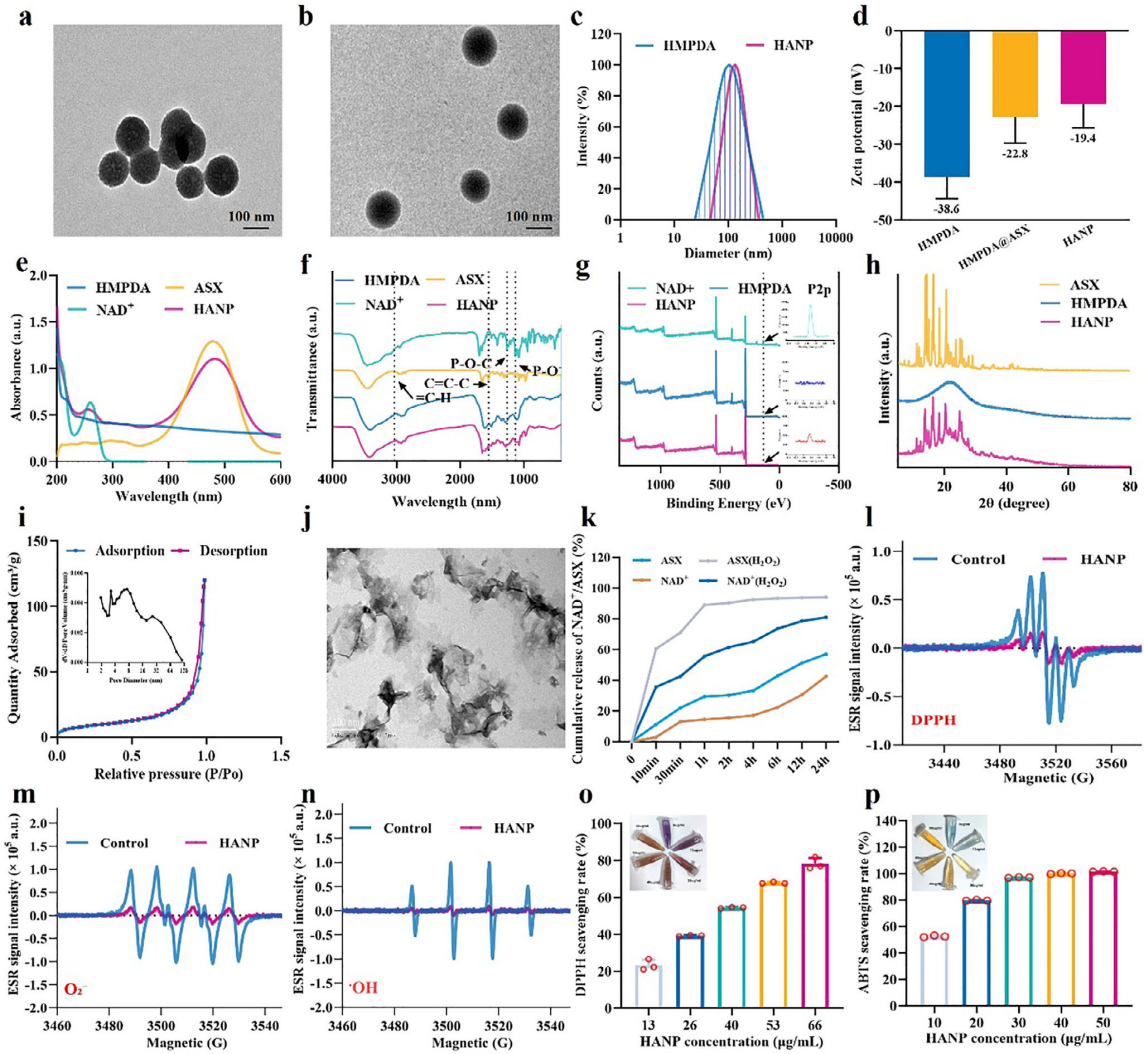
Physicochemical characterization and antioxidant properties of HANP. (a, b) TEM images of HMPDA and HANP. (c, d) Particle size distribution (DLS) and Zeta potential. (e–h) Compositional analysis via UV–vis (e), FTIR (f), XPS (g), and XRD (h). (i) Nitrogen adsorption–desorption isotherms. (j) TEM images of HANP degradation exposure to 100 µm H_2_O_2_ under acidic conditions. (k) Cumulative drug release profiles. (l–p) ESR and efficiency (colorimetric assay) against DPPH, superoxide, and hydroxyl radicals. Data are presented as mean ± s.d.

HMPDA nanoparticles were synthesized via a soft‐templating approach (Figure S3). To enhance colloidal stability and physiological biocompatibility, the surfaces were modified with NH_2_‐PEG‐COOH following the co‐loading of ASX and NAD^+^, yielding HANP. Transmission electron microscopy (TEM) revealed that both HMPDA and HANP maintained uniform spherical structures, with the hollow cavity of HANP appearing partially occupied, indicating successful cargo encapsulation (Figure 2a,b). Dynamic light scattering (DLS) and zeta potential measurements confirmed these structural transitions, as evidenced by a hydrodynamic diameter increase from 103.7 to 132.4 nm and a surface charge shift from −38.6 to −19.4 mV upon drug loading and PEGylation (Figure 2c,d).

The successful co‐loading and molecular integrity of NAD^+^ and ASX were validated through multi‐technique physicochemical analyses. UV–vis and FTIR spectra of HANP retained the characteristic absorption peaks of both therapeutic agents, with new vibrational bands at ∼1050–1100 cm^−^^1^ (P─O─C and P─O^−^) confirming the incorporation of the phosphate groups from NAD^+^ (Figure 2e,f). Specifically, the encapsulation efficiencies of ASX and NAD^+^ were 67.08% and 25.72%, with loading capacities of 24.48% and 46.94%, respectively. XPS further corroborated NAD^+^ loading via the emergence of P2p peaks (Figure 2g). While pure ASX exhibited high crystallinity, XRD patterns suggested that ASX was encapsulated in a predominantly amorphous or partially dispersed state within the dopamine matrix (Figure 2h). Both HMPDA and HANP exhibited Type IV isotherms and H3 hysteresis loops, characteristic of a robust mesoporous structure with pores ranging from 2 to 50 nm. The consistency of these profiles before and after loading confirms that the carrier's structural integrity is preserved, providing ample pore volume for the synergistic delivery of NAD^+^ and ASX (Figure 2i and Figure S4).

Owing to the ROS‐sensitive nature of the dopamine backbone, HANP exhibited distinct stimulus‐responsive behavior. While structural integrity was maintained under physiological pH, exposure to H_2_O_2_ induced marked disintegration and triggered accelerated cargo release, with over 90% of ASX and ∼70% of NAD^+^ liberated within 6 h (Figure 2j,k and Figure S5). Beyond its role as a responsive carrier, HANP demonstrated potent intrinsic radical‐scavenging activity. Electron spin resonance (ESR) and colorimetric assays (DPPH and ABTS) revealed a dose‐dependent neutralization of multiple reactive species, including •DPPH, •O_2_^−^, and •OH (Figure 2l–p). HANP exhibited excellent physicochemical stability with minimal changes in size and dispersity under varying storage durations and pH conditions (Figure S6). Collectively, these results support the use of HANP as a stable, ROS‐responsive nanospray that integrates potent antioxidant capacity with targeted drug‐delivery functionality.

### 2.3 HANP Nanospray Shows Preferential Pulmonary Retention and Alleviates RILI

**FIGURE 3:**
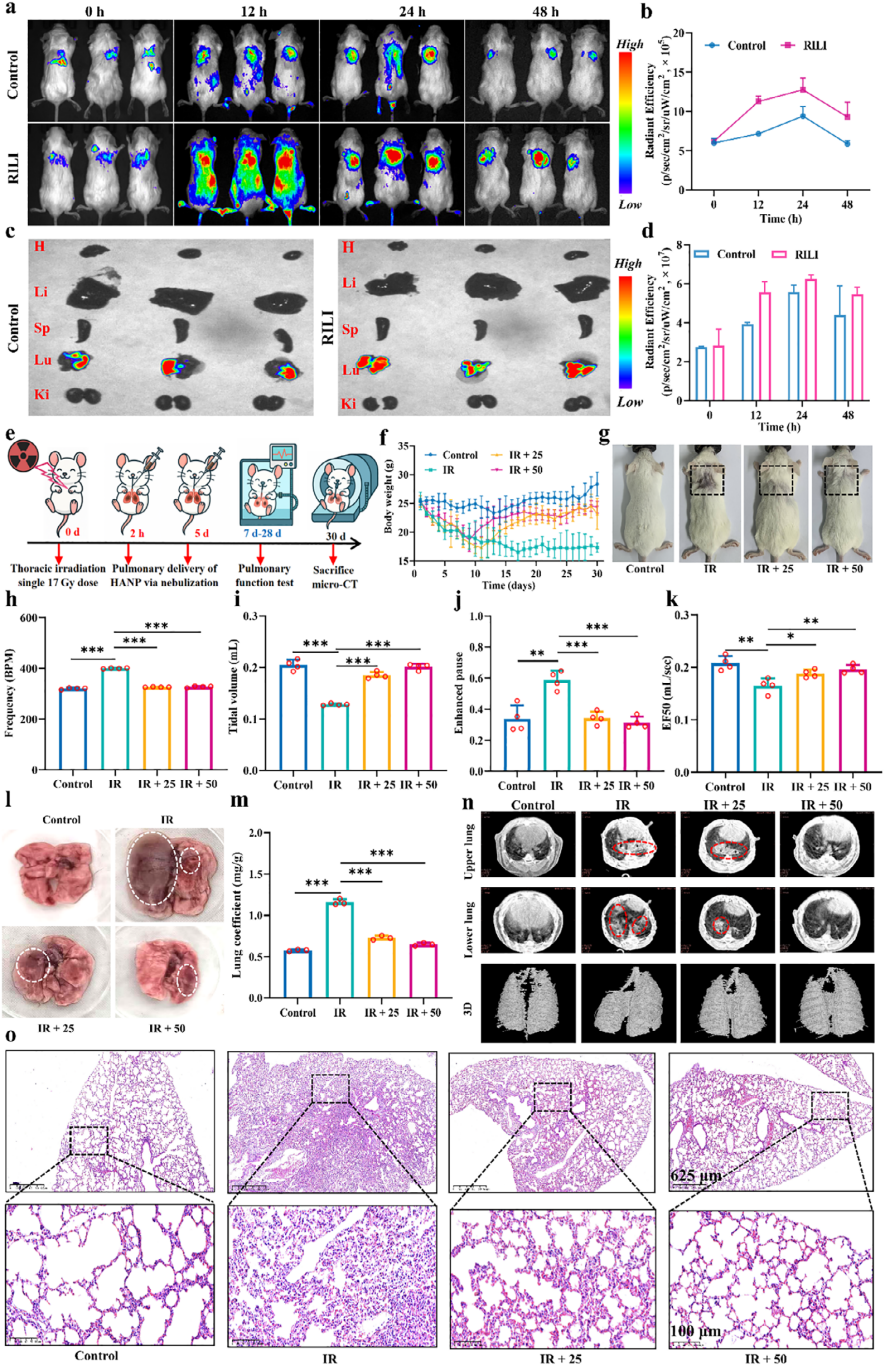
Biodistribution and therapeutic efficacy of HANP in a murine RILI model. (a–d) In vivo and ex vivo fluorescence imaging of ICG@HANP biodistribution over 48 h (H: heart; Li: liver; Sp: spleen; Lu: lung; Ki: kidney) (*n* = 3). (e) Schematic of the treatment protocol. (f, g) Body weight monitoring and gross appearance (*n* = 5). (h–k) Pulmonary function assessment (frequency, tidal volume, Penh, EF_50_) at day 28 (*n* = 4). (l–n) Lung structural evaluation via gross morphology (l), lung coefficient (m) (*n* = 3), and micro‐CT imaging (n). (o) H&E staining of lung sections. Data are presented as mean ± s.d. Statistical analysis was performed using one‐way ANOVA with Tukey's post hoc test. *p < 0.05, **p < 0.01, ***p < 0.001.

Prior to therapeutic evaluation, the pulmonary distribution of HANP nanospray was assessed using indocyanine green‐loaded nanoparticles (ICG@HANP). Laser diffraction analysis showed that nebulized HANP generated respirable droplets with D10 = 2.93 µm, D50 = 6.36 µm, and D90 = 14.06 µm, suitable for lower respiratory tract deposition (Figure S7). The mucus penetration assay showed that HANP traversed the mucus layer in a time‐dependent manner. HANP was initially located above the mucus layer and gradually penetrated through the barrier over time, which was further supported by the quantitative penetration analysis (Figure S8). In vivo fluorescence imaging revealed that signals were predominantly localized in the thoracic region, with peak intensity observed at 24 h post‐nebulization (Figure 3a,b). Notably, fluorescence retention was consistently higher in RILI mice compared to healthy controls, suggesting enhanced accumulation of HANP within inflamed pulmonary tissue. Ex vivo imaging confirmed that fluorescence was confined primarily to the lungs, with minimal off‐target distribution to other major organs (Figure 3c,d), thereby validating the efficacy of nebulization for targeted lung delivery. The fluorescence signal was broadly distributed throughout the lung parenchyma rather than being confined to the proximal airways, supporting effective delivery to broad lung regions, including distal lung areas (Figure S9). Collectively, these findings indicate that HANP is a suitable candidate for nebulized pulmonary delivery, featuring appropriate aerosolization, effective mucus penetration, distal lung deposition, and preferential accumulation in inflamed lung tissue.

The therapeutic potential of HANP was subsequently evaluated in a murine model of RILI induced by single‐dose thoracic irradiation (17 Gy) (Figure 3e). While the IR group exhibited rapid and sustained body weight loss, HANP treatment promoted dose‐dependent recovery, with the 50 mg/kg group restoring body weight to near‐control levels by day 30 (Figure 3f). This systemic improvement was accompanied by enhanced physical condition, including smoother fur texture and increased locomotor activity (Figure 3g). Pulmonary function, assessed via whole‐body plethysmography, was significantly impaired in IR mice—characterized by reduced lung volumes and elevated airway resistance—but was markedly restored following HANP treatment (Figure 3h–k and Figures S10–S13).

Morphological and histological assessments further corroborated these functional benefits. Gross examination and lung coefficient analysis revealed that HANP treatment effectively mitigated radiation‐induced pulmonary edema and structural deterioration (Figure 3l,m). Micro‐CT imaging demonstrated that HANP preserved lung parenchymal architecture, attenuating consolidation and fibrotic‐like lesions observed in the IR group (Figure 3n). Histologically, H&E staining confirmed that while irradiation caused severe alveolar wall thickening and inflammatory infiltration, HANP administration (50 mg/kg) preserved alveolar integrity with minimal inflammation, closely resembling healthy lung tissue (Figure 3o). Taken together, these findings demonstrate that HANP nanospray confers robust, dose‐dependent protection against radiation‐induced pulmonary injury.

### 2.4 HANP Attenuates Radiation‐Induced Apoptosis, Oxidative Stress, and Inflammatory Responses

**FIGURE 4:**
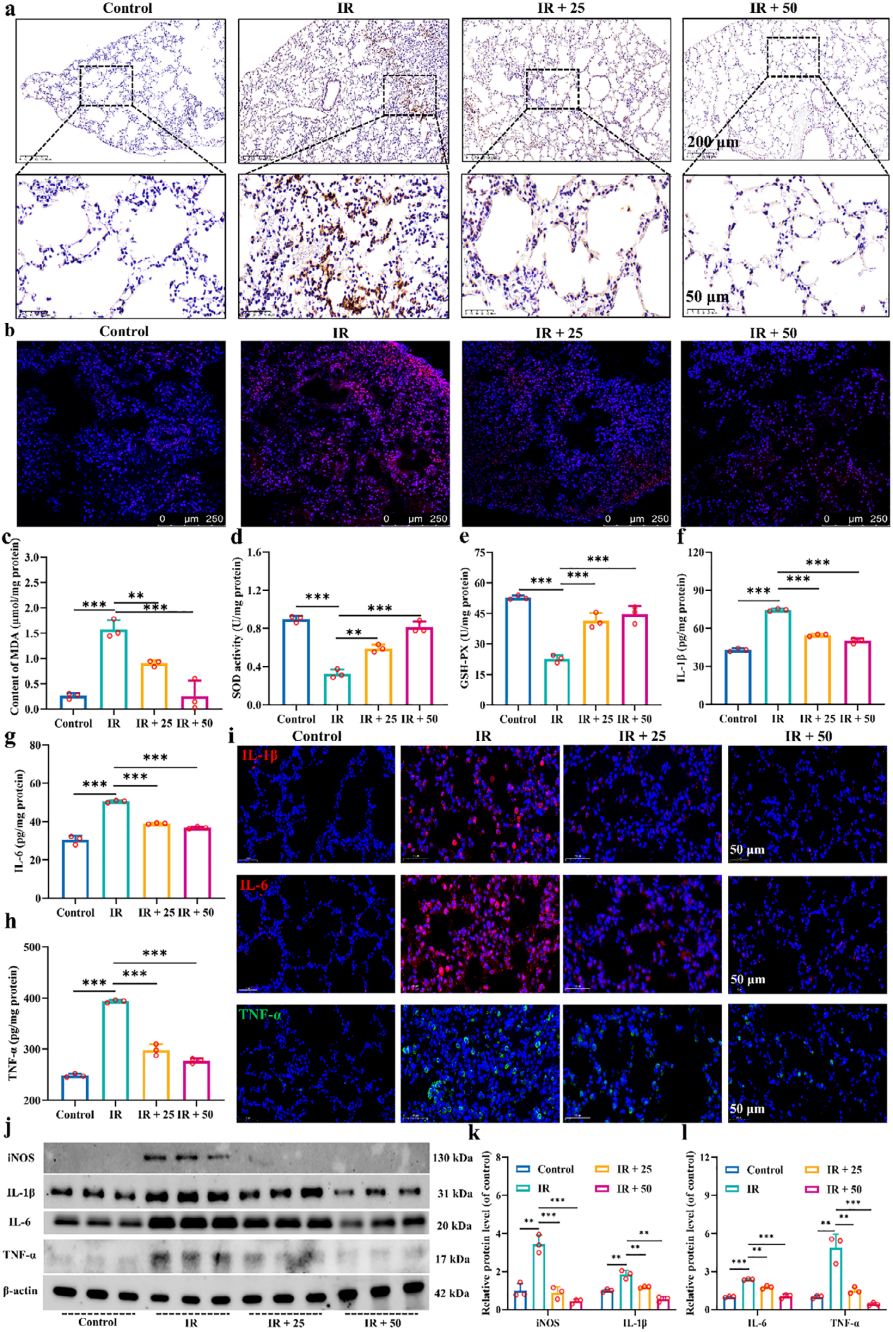
Assessment of apoptosis, oxidative stress, and inflammation in lung tissues. (a) TUNEL staining of apoptotic cells. Scale bars: upper panels, 200 µm; lower panels, 50 µm. (b) DHE staining for ROS levels, Scale bar: 250 µm. (c–e) Quantification of oxidative stress markers (MDA, SOD, GSH‐Px) (*n* = 3). (f–i) Inflammatory cytokine levels (IL‐1β, IL‐6, TNF‐α) measured by ELISA (f–h) (*n* = 3) and immunofluorescence (i). (j–l) Western blot analysis of inflammatory markers (*n* = 3). Data are presented as mean ± s.d. Statistical analysis was performed using one‐way ANOVA with Tukey's post hoc test. *p < 0.05; **p < 0.01; ***p < 0.001.

To elucidate the cellular mechanisms driving tissue recovery, the impact of HANP on apoptosis, oxidative stress, and inflammatory responses was evaluated. TUNEL staining revealed extensive cellular apoptosis in the IR group, which was significantly mitigated by HANP treatment, particularly at 50 mg/kg, restoring levels comparable to controls (Figure 4a). Parallel assessment of redox status confirmed the antioxidant efficacy of HANP. Dihydroethidium (DHE) staining indicated intense ROS accumulation in irradiated lungs, a phenomenon effectively suppressed by HANP in a dose‐dependent manner (Figure 4b). Biochemical analysis further corroborated this rebalancing: HANP treatment significantly reduced malondialdehyde (MDA) levels—a marker of lipid peroxidation—while restoring the depleted activities of superoxide dismutase (SOD) and glutathione peroxidase (GSH‐Px) (Figure 4c–e).

Given the reciprocal relationship between oxidative stress and inflammation, the impact of HANP on pro‐inflammatory mediators was subsequently examined. ELISA quantification demonstrated that irradiation significantly elevated pulmonary levels of IL‐1β, IL‐6, and TNF‐α. Notably, HANP treatment dose‐dependently suppressed the secretion of these cytokines (Figure 4f–h). This anti‐inflammatory effect was further validated at the protein level by immunofluorescence staining and Western blot analysis, which showed a marked downregulation of iNOS, IL‐1β, IL‐6, and TNF‐α in HANP‐treated lung tissues compared to the IR group (Figure 4i–l). Overall, these data indicate that HANP preserves lung homeostasis by attenuating apoptosis, oxidative stress, and inflammatory responses.

### 2.5 HANP Activates a PPAR‐γ‐Associated Transcriptional Program and Shifts Pulmonary Macrophages Toward an M2‐Like Phenotype

**FIGURE 5:**
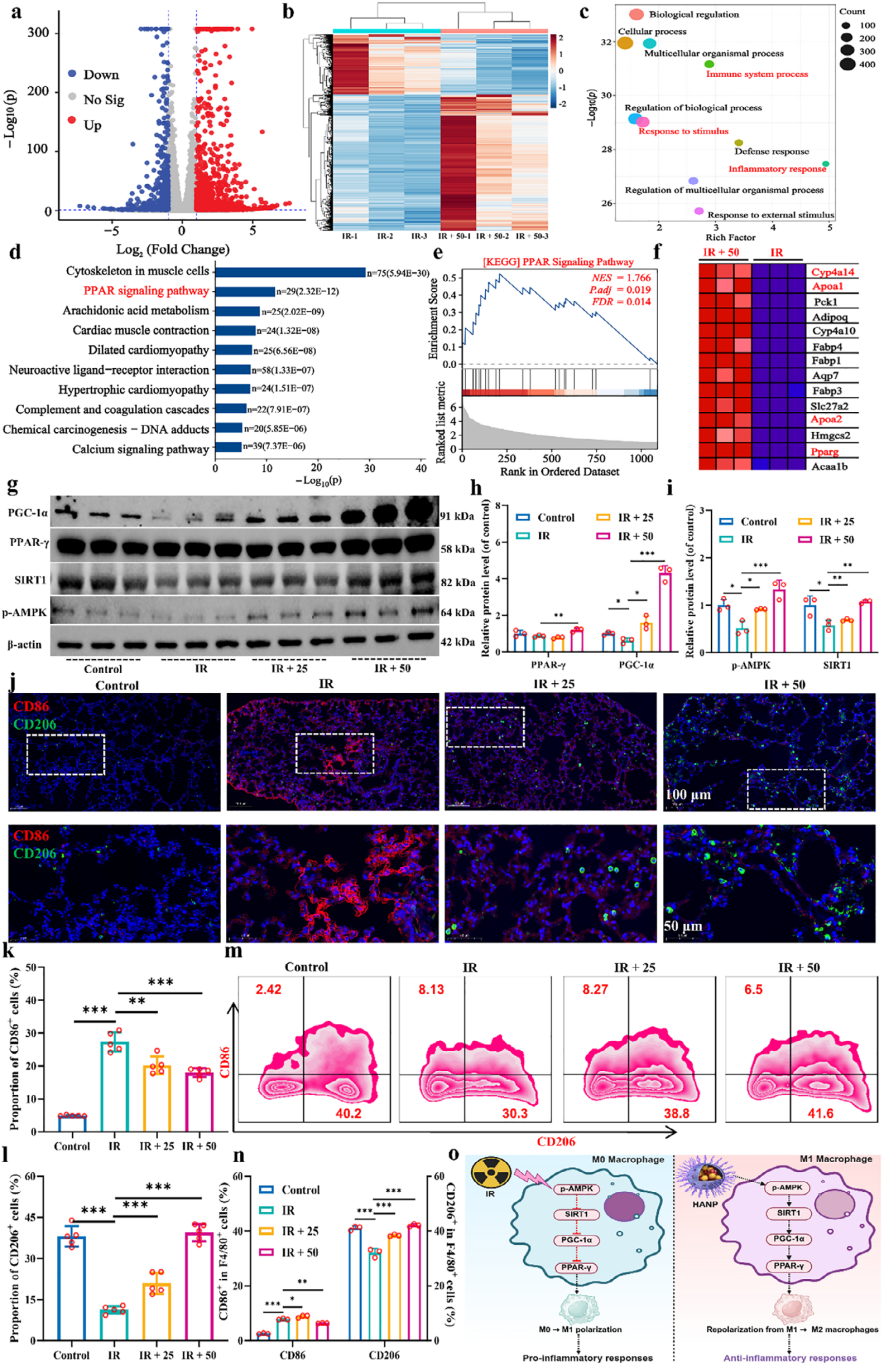
HANP modulates macrophage polarization via the PPAR signaling pathway in vivo. (a–e) Transcriptomic analysis of HANP‐treated lung tissues, including Volcano plot (a), clustering heatmap (b), KEGG enrichment (c, d), and GSEA of PPAR signaling (e). (f) Heatmap of PPAR pathway genes. (g–i) Western blot validation of the SIRT1–AMPK–PGC‐1α–PPAR‐γ axis in lung tissues (*n* = 3). (j–n) Evaluation of M1 (CD86^+^) and M2 (CD206^+^) polarization via immunofluorescence (j–l) (*n* = 5) and flow cytometry (m, n) (*n* = 3). (o) Proposed mechanism by which HANP alleviates RILI through the SIRT1–AMPK–PGC‐1α–PPAR‐γ signaling axis. Data are presented as mean ± s.d. Statistical analysis was performed using one‐way ANOVA with Tukey's post hoc test. *p < 0.05; **p < 0.01; ***p < 0.001.

Transcriptomic profiling was performed on lung tissues to elucidate the molecular mechanisms driving HANP‐mediated protection. Volcano plot analysis identified 1615 differentially expressed genes (DEGs) between the IR and HANP‐treated groups (Figure 5a). Hierarchical clustering revealed distinct transcriptional signatures, indicating substantial reprogramming of the pulmonary transcriptome (Figure 5b). Gene Ontology (GO) enrichment analysis demonstrated that HANP treatment significantly downregulated biological processes associated with immune activation and inflammatory responses (Figure 5c). Notably, Gene Set Enrichment Analysis (GSEA) and KEGG enrichment highlighted the PPAR signaling pathway as one of the major pathways associated with HANP treatment, with key regulators of lipid metabolism and antioxidant defense, including *Pparg*, *Apoa1*, and *Cyp4a14*, being upregulated (Figure 5d–f).

The activation of PPAR‐γ and its upstream regulators was further validated at the protein level. Interestingly, irradiation alone did not significantly alter the protein expression of PPAR‐γ. While low‐dose HANP (25 mg/kg) treatment showed no obvious effect on PPAR‐γ, the high dose (50 mg/kg) significantly upregulated its expression relative to the IR group. Conversely, irradiation significantly suppressed the expression of PGC‐1α, which was effectively reversed by HANP treatment, showing a marked increase in a dose‐dependent manner (Figure 5g,h). Furthermore, irradiation decreased the levels of the upstream regulators SIRT1 and phosphorylated AMPK (p‐AMPK). HANP administration significantly upregulated both SIRT1 and p‐AMPK, with protein levels in the high‐dose group even surpassing those of the control group (Figure 5g–i). These findings suggest that although irradiation does not notably alter the protein expression of PPAR‐γ, it likely impairs its functional activity by suppressing its upstream regulators and transcriptional coactivator (PGC‐1α). HANP treatment was therefore associated with reactivation of the AMPK/SIRT1/PGC‐1α/PPAR‐γ signaling axis.

Consistent with the potentiation of PPAR‐γ signaling, HANP modulated macrophage polarization phenotypes within the lung tissue. Single‐cell transcriptomic data indicated that PPAR‐γ expression is primarily localized to macrophages. Immunofluorescence staining demonstrated that irradiation induced a shift toward a pro‐inflammatory M1 phenotype (CD86^+^), whereas HANP treatment dose‐dependently promoted an anti‐inflammatory M2 phenotype (CD206^+^) (Figure 5j–l). This observation was corroborated by flow cytometric analysis: irradiation increased the proportion of M1 macrophages from 2.42% to 8.13%, while decreasing M2 macrophages from 40.2% to 30.3%. HANP administration (50 mg/kg) effectively reversed this trend, reducing M1 cells to 6.5% and elevating M2 cells to 41.6% (Figure 5m,n and Figure S14). Collectively, these in vivo data indicate that HANP reprograms pulmonary macrophages toward a reparative M2 phenotype, concurrent with the robust potentiation of PPAR‐γ signaling.

**FIGURE 6:**
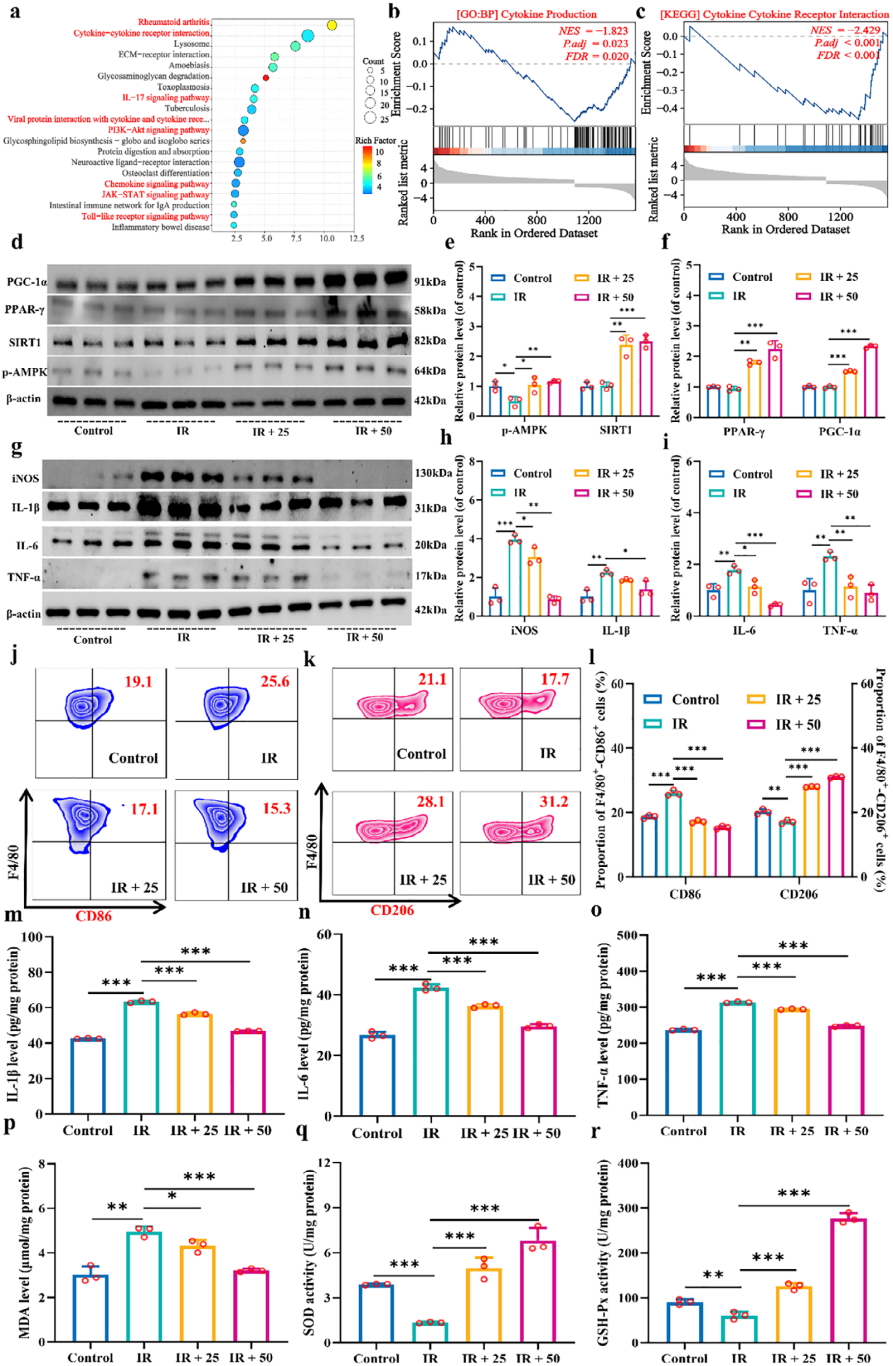
In vitro validation of the anti‐inflammatory and antioxidant mechanism in macrophages. (a–c) Enrichment analysis of inflammation‐related pathways. (d–f) Western blot analysis of the SIRT1–AMPK–PGC‐1α–PPAR‐γ axis in irradiated macrophages (*n* = 3). (g–i) Expression of pro‐inflammatory mediators (*n* = 3). (j–l) Flow cytometric analysis of M1/M2 phenotypes (*n* = 3). (m–r) Quantification of secreted cytokines (ELISA) and oxidative stress markers (MDA, SOD, GSH‐Px) (*n* = 3). Data are presented as mean ± s.d. Statistical analysis was performed using one‐way ANOVA with Tukey's post hoc test. *p < 0.05; **p < 0.01; ***p < 0.001.

Beyond the modulation of PPAR signaling, KEGG enrichment analysis highlighted that HANP broadly suppressed inflammation‐associated pathways, including cytokine–cytokine receptor interaction, IL‐17 signaling, and Toll‐like receptor signaling (Figure 6a). Gene Set Enrichment Analysis (GSEA) further confirmed strong negative enrichment for gene sets related to cytokine production in HANP‐treated tissues (Figure 6b,c). To functionally validate these transcriptional findings, an in vitro model using primary peritoneal macrophages was established (Figure S15). HANP exhibited excellent biocompatibility across a broad concentration range (Figure S16) and significantly attenuated radiation‐induced cellular injury. Specifically, HANP treatment preserved cell viability, reduced intracellular ROS accumulation, and prevented radiation‐induced calcium overload and mitochondrial membrane potential loss (Figures S17–S20). Furthermore, HANP effectively alleviated DNA damage, as evidenced by reduced γ‐H2AX foci formation (Figure S21).

The impact of HANP on the SIRT1–AMPK–PGC‐1α–PPAR‐γ signaling axis was subsequently evaluated at the cellular level. Western blot analysis showed that irradiation suppressed the expression of SIRT1, p‐AMPK, PGC‐1α, and PPAR‐γ (Figure 6d). In contrast, HANP treatment significantly upregulated these proteins in a dose‐dependent manner. Compared to the IR group, the expression levels of SIRT1, p‐AMPK, PGC‐1α, and PPAR‐γ in the high‐dose HANP group were increased by approximately 1.8‐, 2.1‐, 2.5‐, and 2.2‐fold, respectively (Figure 6e,f). This robust upregulation indicates that HANP acts as a potent activator of the SIRT1–AMPK–PGC‐1α–PPAR‐γ signaling axis.

Concurrently, macrophage polarization phenotypes were assessed to link signaling activation with functional outcomes. Western blot analysis demonstrated that irradiation induced the upregulation of classical M1 markers (iNOS, IL‐1β, IL‐6, TNF‐α), which were significantly suppressed by HANP treatment (Figure 6g–i). Flow cytometry provided quantitative confirmation of this phenotypic shift: while irradiation increased the proportion of M1 macrophages (F4/80^+^CD86^+^) to 25.6%, HANP treatment (50 µg/mL) effectively reduced this population to 15.3%. Conversely, the proportion of M2 macrophages (F4/80^+^CD206^+^), which was suppressed by irradiation (17.7%), was significantly expanded by HANP treatment to 31.2% (Figure 6j–l). This anti‐inflammatory profile was further corroborated by ELISA, which showed dose‐dependent reductions in the secretion of IL‐1β, IL‐6, and TNF‐α, with levels in the high‐dose group comparable to controls (Figure 6m–o).

Finally, the effect of HANP on macrophage redox homeostasis was quantified. Irradiated macrophages exhibited oxidative imbalance, characterized by elevated lipid peroxidation (MDA) and compromised antioxidant enzyme activities. HANP treatment effectively mitigated these alterations; specifically, MDA levels were reduced to near‐control baselines, while the activities of SOD and GSH‐Px were significantly enhanced, surpassing control levels (Figure 6p–r). Overall, these in vitro findings demonstrate that HANP protects macrophages against radiation‐induced injury and reprograms them toward an anti‐inflammatory M2 phenotype, a process driven by the potentiation of the SIRT1–AMPK–PGC‐1α–PPAR‐γ axis and the restoration of redox homeostasis.

### 2.6 HANP Restores Mitochondrial Homeostasis by Promoting Mitophagy and Mitochondrial Biogenesis

**FIGURE 7:**
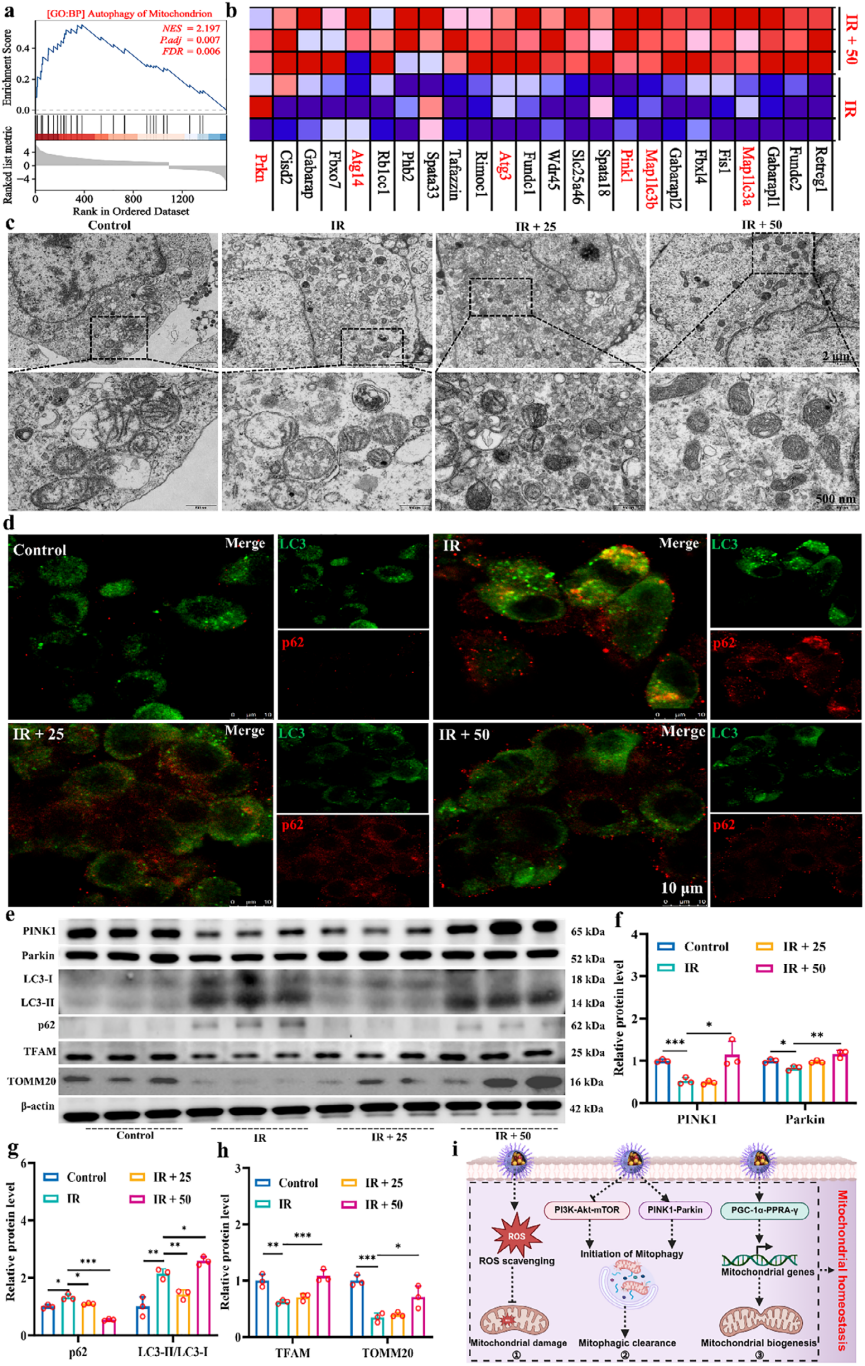
HANP promotes mitophagy and mitochondrial biogenesis to preserve mitochondrial homeostasis in irradiated macrophages. (a, b) Transcriptomic analysis of mitophagy‐related genes in lung tissues via GSEA (a) and heatmap (b). (c) TEM visualization of mitochondrial ultrastructure. Scale bars: upper panels, 2 µm; lower panels, 500 nm. (d) Immunofluorescence staining of LC3 (green) and p62 (red) in macrophages. Scale bars: 10 µm. (e–h) Western blot analysis (e) and quantification (f–h) of mitophagy and biogenesis markers (PINK1, Parkin, LC3, p62, TFAM, TOMM20) (*n* = 3). (i) Proposed mechanism of HANP‐mediated mitochondrial quality control. Data are presented as mean ± s.d. Statistical analysis was performed using one‐way ANOVA with Tukey's post hoc test. *p < 0.05; **p < 0.01; ***p < 0.001.

Given that the SIRT1–AMPK–PGC‐1α axis is a master regulator of mitochondrial homeostasis, transcriptomic data were further analyzed to assess mitochondrial quality control mechanisms. GSEA revealed a significant enrichment of the GO term “autophagy of mitochondrion” (GO:0000422), with a pronounced negative enrichment score in the IR group, indicating suppression of mitophagy‐associated genes. In contrast, HANP treatment markedly upregulated these genes, including key regulators of selective autophagic clearance such as *Pink1, Prkn (Parkin)*, *Map1lc3a*, *Map1lc3b*, and *Atg14* (Figure 7a,b). Ultrastructural examination further corroborated these transcriptional findings. TEM demonstrated that irradiation induced extensive mitochondrial damage—characterized by swelling, cristolysis, and vacuolization—alongside the accumulation of autophagosomes, indicative of a blockade in autophagic flux. HANP treatment effectively preserved mitochondrial morphology and reduced the burden of accumulated autophagosomes in a dose‐dependent manner (Figure 7c). To assess autophagic flux functionally, immunofluorescence staining for LC3 and p62 was performed. In the IR group, the prominent co‐accumulation of LC3 and p62 signaled impaired autophagic degradation. HANP treatment significantly reduced both signals and their co‐localization, suggesting the restoration of efficient autophagic flux (Figure 7d).

The molecular mechanisms driving this restoration were subsequently dissected. Western blot analysis revealed that irradiation downregulated the mitophagy initiators PINK1 and Parkin while elevating p62 and the LC3‐II/I ratio, confirming suppressed mitophagy. HANP treatment dose‐dependently upregulated PINK1 and Parkin expression and reduced p62 accumulation, consistent with the reactivation of PINK1–Parkin‐mediated mitophagy (Figure 7e–g). Furthermore, KEGG pathway analysis highlighted a significant downregulation of the PI3K–AKT signaling pathway—a known negative regulator of autophagy—following HANP treatment (Figure 6a). Validation by western blot confirmed that HANP suppressed the radiation‐induced activation of PI3K, AKT, and mTOR, thereby relieving the inhibition on autophagy (Figure S22).

Beyond clearance, the impact of HANP on mitochondrial biogenesis was evaluated. Given the activation of the SIRT1–AMPK–PGC‐1α–PPAR‐γ axis described earlier, we examined the expression of canonical biogenesis markers. Irradiation markedly reduced the levels of TFAM (Mitochondrial Transcription Factor A) and TOMM20 (Translocase of Outer Mitochondrial Membrane 20), indicating compromised mitochondrial renewal. Notably, HANP treatment significantly potentiated the expression of both markers in a dose‐dependent manner, with levels in the high‐dose group surpassing those of controls (Figure 7e–h). Collectively, these findings demonstrate that HANP maintains mitochondrial homeostasis by orchestrating a dual mechanism: facilitating the clearance of damaged mitochondria via PINK1–Parkin‐mediated mitophagy and promoting the generation of new mitochondria through PGC‐1α–PPAR‐γ‐driven biogenesis.

To further determine whether the mechanistic effects observed in thioglycolate‐elicited peritoneal macrophages could be reproduced in an alveolar macrophage‐relevant model, we performed additional validation experiments in the murine alveolar macrophage cell line MH‐S. Flow cytometric analysis showed that irradiation increased the proportion of CD86‐positive cells and reduced the CD206‐positive population, indicating a shift toward a pro‐inflammatory phenotype (Figure S23a,b). HANP Treatment reversed these changes, as evidenced by a reduced CD86‐positive population and an increased proportion of CD206‐positive cells. Western blot analysis further showed that irradiation suppressed the expression of PPAR‐γ, PGC‐1α, and SIRT1, whereas HANP treatment restored their expression to varying extents. In parallel, irradiation induced marked alterations in autophagy/mitophagy‐associated proteins, including PINK1, Parkin, and p62, which were substantially normalized following treatment. Moreover, HANP treatment also modulated the expression of TFAM and TOMM20 in irradiated MH‐S cells, supporting an effect on mitochondrial biogenesis and mitochondrial homeostasis (Figure S23c–f). Collectively, these findings provide additional evidence that the HANP intervention regulates macrophage polarization and mitochondrial quality‐control‐related pathways in an alveolar macrophage‐relevant setting.

Building on the irradiation‐induced suppression of PPAR‐γ signaling described above, we next evaluated the effect of HNAP on this pathway in MH‐S cells. HNAP treatment enhanced PPAR‐γ signaling in irradiated MH‐S cells, as evidenced by increased PPAR‐γ expression and a more pronounced nuclear accumulation pattern in immunofluorescence analysis (Figure S24a). More importantly, HNAP markedly restored PPAR‐γ transcription factor activity, indicating functional reactivation of this pathway after irradiation (Figure S24b). Consistent with the recovery of PPAR‐γ activity, the expression of its downstream targets CD36 and Arg1 was also significantly elevated following HNAP treatment, with the higher dose showing a stronger restorative effect (Figure S24c–e). These findings suggest that HNAP rescues the irradiation‐impaired PPAR‐γ axis at multiple levels, including protein expression, nuclear translocation, and transcriptional output.

### 2.7 Pharmacological Inhibition of PPAR‐γ Abolishes HANP‐Mediated Protection Against RILI

**FIGURE 8:**
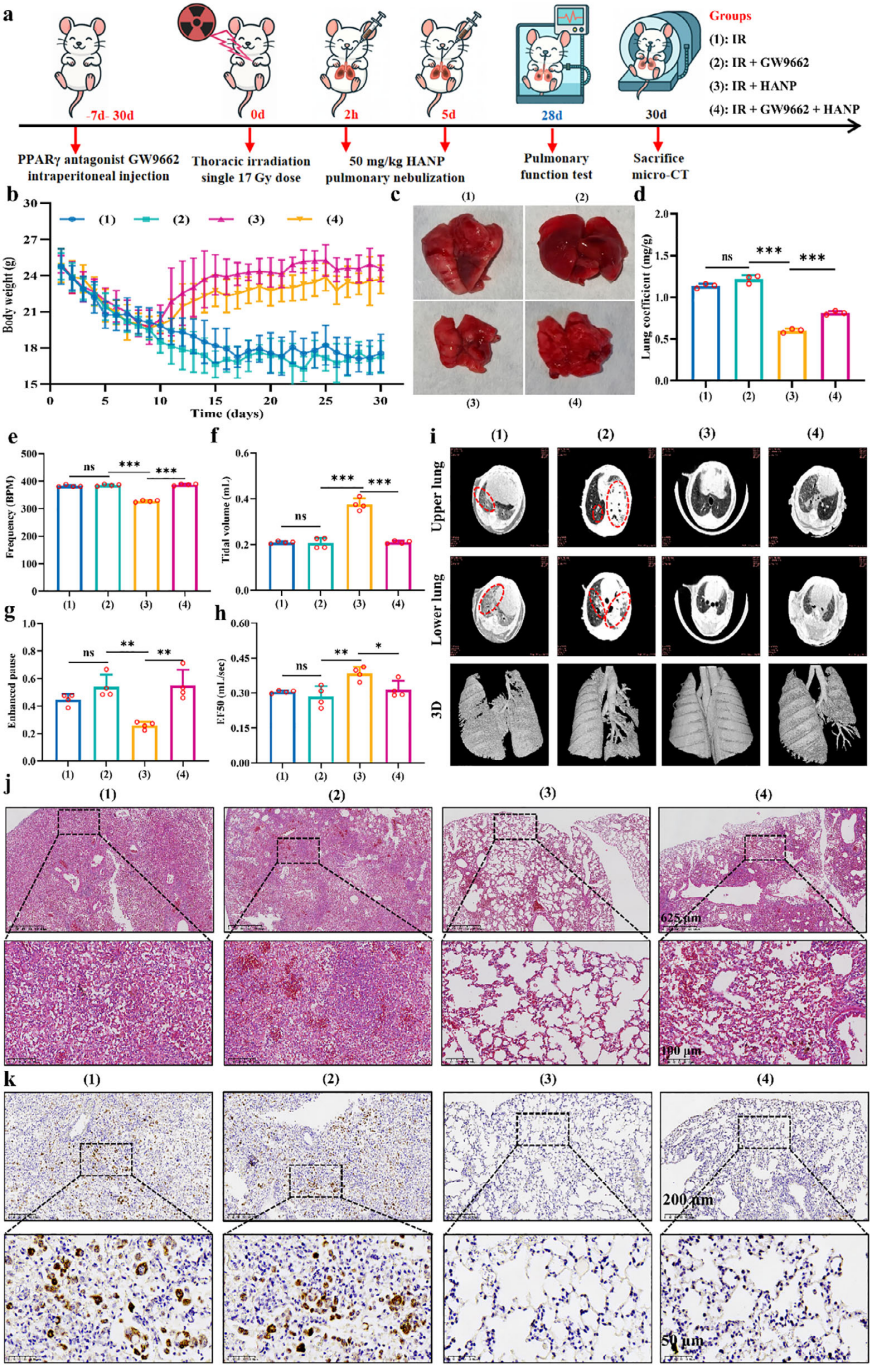
PPAR‐γ is required for HANP‐mediated lung protection against RILI. (a) Schematic of the RILI mouse model (17 Gy ^6^ ^0^Co γ‐irradiation) and treatment schedule. (b–d) Evaluation of body weight changes (b) (*n* = 8), gross lung morphology (c), and lung coefficient (d) post‐irradiation (*n* = 3). (e–h) Pulmonary function parameters measured on day 28 (*n* = 4): respiratory frequency (e), tidal‐volume loss (f), enhanced pause (Penh) (g), and elastance (h). (i) Micro‐CT axial sections (top/middle) and 3D reconstructions (bottom). Red circles indicate lesion areas. (j, k) Histological assessment via H&E (j) and TUNEL staining (k) of lung tissues. Data are presented as mean ± s.d. Statistical analysis was performed using one‐way ANOVA with Tukey's post hoc test. *p < 0.05; **p < 0.01; ***p < 0.001. Scale bars: 500 µm (upper) and 100 µm (lower) in (j); 200 µm (upper) and 50 µm (lower) in (k). Groups: IR, IR + GW9662, IR + HANP, IR + GW9662 + HANP.

To delineate the functional requirement of PPAR‐γ in HANP‐mediated therapy, a pharmacological intervention study was conducted using GW9662, a selective irreversible PPAR‐γ antagonist (Figure 8a). Mice subjected to thoracic irradiation were treated with HANP, with or without GW9662 co‐administration. HANP treatment significantly mitigated radiation‐induced weight loss and pulmonary edema; however, these protective effects were largely abrogated by GW9662 (Figure 8b–d). Pulmonary function tests corroborated these findings: the improvements in tidal volume and EF_50_ conferred by HANP were markedly attenuated when PPAR‐γ activity was inhibited (Figure 8e–h and Figure S25). Structurally, micro‐CT imaging and H&E staining revealed that HANP preserved alveolar integrity and reduced consolidation, whereas GW9662 co‐treatment negated these benefits, resulting in severe architectural disruption comparable to or exceeding that of the IR group (Figure 8i,j). Furthermore, the anti‐apoptotic effect of HANP, as evidenced by reduced TUNEL staining, was significantly reversed by GW9662 (Figure 8k). Collectively, these data indicate that PPAR‐γ signaling is indispensable for the structural and functional protection provided by HANP against RILI.

### 2.8 PPAR‐γ Governs Macrophage Polarization and Mitochondrial Biogenesis but is Dispensable for HANP‐Induced Mitophagy

**FIGURE 9:**
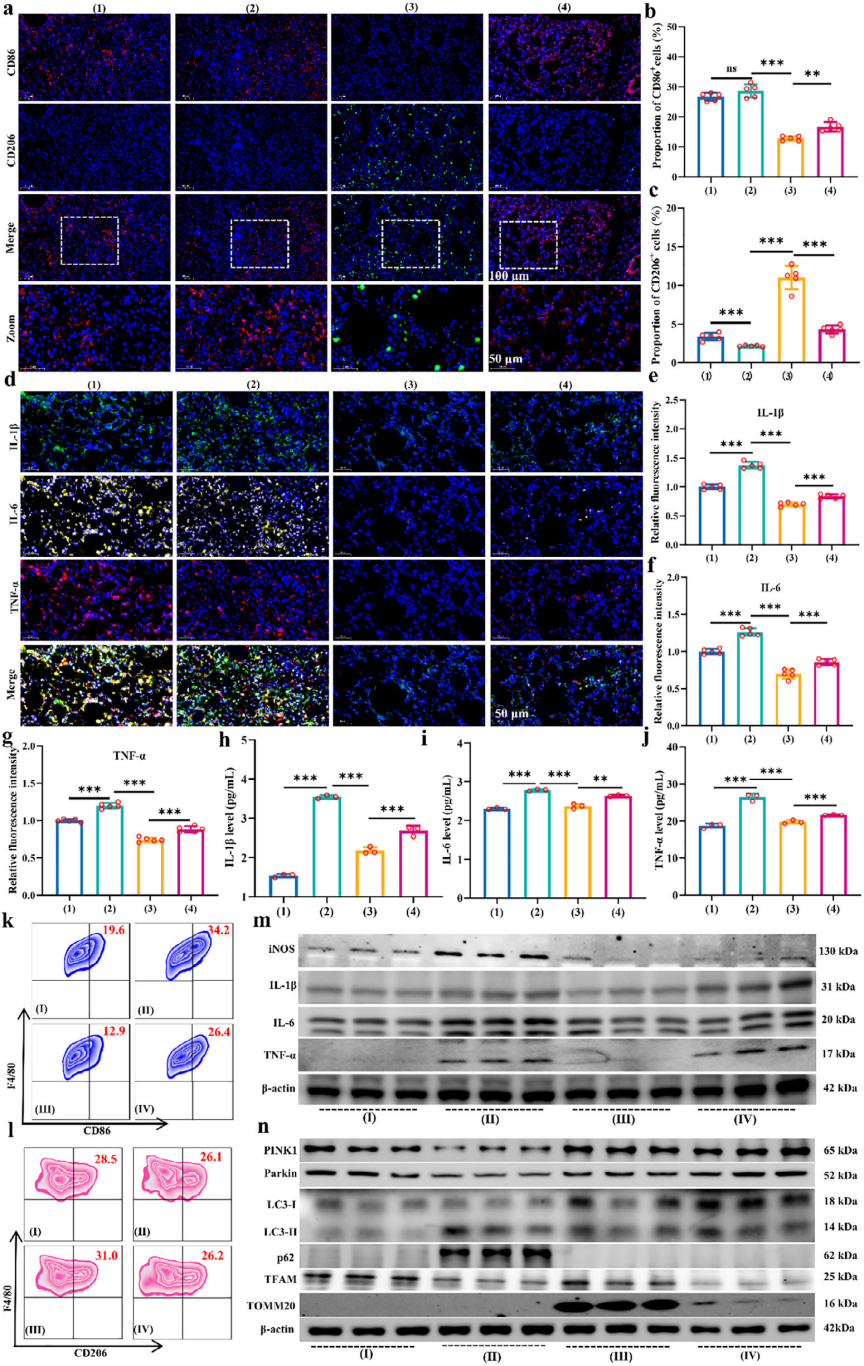
PPAR‐γ mediates the anti‐inflammatory and immunomodulatory effects of HANP in RILI. (a–c) Immunofluorescence staining (a) and quantification (b, c) of CD86^+^ (M1, red) and CD206^+^ (M2, green) macrophages in lung tissues (*n* = 5). (d–g) Immunofluorescence (d) and quantitative analysis (e–g) of pro‐inflammatory cytokines (IL‐1β, IL‐6, TNF‐α) in lung tissues (*n* = 5). (h–j) ELISA quantification of IL‐1β, IL‐6, and TNF‐α secreted in macrophage supernatants (*n* = 3). (k, l) Flow cytometric analysis of CD86^+^/F4/80^+^ (M1) and CD206^+^/F4/80^+^ (M2) macrophage populations. (m, n) Western blot analysis of pro‐inflammatory markers (m) and mitophagy/mitochondrial biogenesis‐related proteins (n) in macrophages (*n* = 3). Data are presented as mean ± s.d. Statistical analysis was performed using one‐way ANOVA followed by Tukey's post hoc test for multiple comparisons. *p < 0.05, **p < 0.01, ***p < 0.001. Scale bars: upper panels 100 µm, lower panels 50 µm in (a) and (d). Groups: (1): IR, (2): IR + GW9662, (3): IR + HANP, (4): IR + GW9662 + HANP; (I): Control, (II): IR, (III): IR + HANP, (IV): IR + GW9662 + HANP.

The dependency of HANP‐mediated macrophage reprogramming on PPAR‐γ was further evaluated. Immunofluorescence staining demonstrated that HANP treatment shifted the macrophage population from a pro‐inflammatory M1 phenotype (CD86^+^) to an anti‐inflammatory M2 phenotype (CD206^+^). Crucially, this phenotypic shift was blocked by GW9662, which restored CD86 expression and suppressed CD206 levels (Figure 9a–c). Consistent with this, the HANP‐mediated suppression of pro‐inflammatory cytokines (IL‐1β, IL‐6, and TNF‐α) was partially reversed in the presence of GW9662 (Figure 9d–j). Flow cytometric analysis of primary macrophages confirmed these in vivo observations: GW9662 treatment significantly antagonized the HANP‐induced reduction in M1 macrophages (12.9%–26.4%) and the increase in M2 macrophages (31.0%–26.2%) (Figure 9k,l). We next dissected the role of PPAR‐γ in mitochondrial quality control. Western blot analysis revealed divergent regulatory mechanisms. Regarding mitochondrial biogenesis, HANP upregulated the expression of TFAM and TOMM20; however, this upregulation was significantly attenuated by GW9662, indicating that HANP‐driven mitochondrial biogenesis is PPAR‐γ‐dependent (Figure 9m,n). In sharp contrast, the HANP‐induced activation of mitophagy (indicated by PINK1, Parkin, LC3‐II/I, and p62 levels) and the suppression of the PI3K–AKT–mTOR pathway were unaffected by GW9662 co‐treatment (Figure 9n and Figures S26–S28). These findings suggest a dual mechanism of action: HANP promotes mitochondrial biogenesis and M2 polarization via a PPAR‐γ‐dependent pathway, while facilitating mitophagy and inhibiting PI3K–AKT signaling through a PPAR‐γ‐independent mechanism.

### 2.9 HANP Attenuates Late‐Stage Radiation‐Induced Pulmonary Fibrosis

In light of the progressive nature of radiation‐induced pulmonary fibrosis, we further extended the observation period and assessed lung fibrosis at 16 weeks after irradiation. Masson's trichrome staining showed marked collagen deposition and severe structural remodeling in irradiated lungs. In parallel, immunohistochemical staining demonstrated significantly increased α‐SMA, TGF‐β, and Collagen I expression in the irradiation group (Figure S29a). These changes were substantially attenuated by HANP treatment, particularly in the high‐dose group. Consistently, western blot analysis confirmed activation of the TGF‐β/Smad3/α‐SMA signaling pathway in irradiated lungs, which was markedly suppressed following HANP administration (Figure S29b–e). These results support the notion that HANP not only protects against early radiation‐induced lung injury but also exerts a persistent anti‐fibrotic effect during the late phase of injury progression

### 2.10 HANP Mitigates Systemic Toxicity and Promotes Hematopoietic Recovery in a Whole‐Body Irradiation Model

To assess the systemic radioprotective potential of HANP, a lethal whole‐body irradiation model was utilized. Mice exposed to 6 Gy γ‐radiation exhibited severe weight loss and mortality. HANP treatment significantly attenuated radiation‐induced cachexia and improved survival rates, with efficacy comparable to or slightly exceeding that of the clinical radioprotectant Amifostine, particularly in the early post‐irradiation phase (Figure S30). Furthermore, hematological analysis revealed that HANP effectively mitigated radiation‐induced myelosuppression. Peripheral blood counts of white blood cells (WBC), red blood cells (RBC), and platelets (PLT) were significantly preserved in HANP‐treated mice compared to the IR group (Figure S31). These results highlight the potential of HANP as a systemic radioprotectant capable of safeguarding hematopoietic function.

### 2.11 Biocompatibility and Safety Profile of HANP

The physiological safety of HANP was comprehensively evaluated. In healthy mice, HANP administration (25 and 50 mg/kg) caused no significant alterations in lung coefficients or gross pulmonary morphology (Figure S32a). Long‐term monitoring over 30 days showed normal body weight gain and no signs of systemic toxicity (Figure S32b). Hematological parameters (WBC, RBC, PLT) remained within normal physiological ranges throughout the 21‐day observation period, with only a transient, non‐pathological fluctuation in WBC counts on day 7 (Figure S32c–e). Histological examination of major organs (heart, liver, spleen, lung, kidney) revealed no pathological lesions associated with HANP treatment (Figure S33). In addition, Histological examination of the bronchial and bronchiolar regions revealed that treatment‐related abnormalities, including epithelial disruption, obvious mucus accumulation, or discernible structural injury have not been observed under intratracheal administration of HANP. Finally, a hemolysis assay confirmed excellent blood compatibility, with hemolysis rates remaining below 5% across all tested concentrations (Figure S34). Collectively, these data demonstrate that HANP exhibits a favorable safety profile, supporting its potential for clinical translation.

## 3 Discussion

RILI remains a major challenge in thoracic radiotherapy and nuclear emergency settings, and effective clinical countermeasures remain limited [28, 29, 30]. Although current interventions mainly target downstream inflammatory cascades, they often provide incomplete protection, particularly when mitochondrial dysfunction and redox imbalance have already been established [31, 32, 33]. Our integrative multi‐omics analyses identified a macrophage‐associated transcriptional context that may help explain this limited therapeutic responsiveness. Unlike the marked induction of classical pro‐inflammatory mediators [7], PPAR‐γ, a key regulator of immunometabolism and mitochondrial homeostasis, showed no evident compensatory transcriptional activation after irradiation. This finding indicates that in RILI, the failure of endogenous repair is not caused by a lack of receptor machinery, but rather by its suppression within a hostile oxidative microenvironment. Previous studies suggest that irradiation may lead to either upregulation or downregulation of PPAR‐γ expression, depending on the tissue and experimental context [34, 35]. Unlike those reports, our results showed that irradiation did not markedly alter PPAR‐γ expression in pulmonary macrophages, but instead appeared to impair its activity. This difference may be attributable to the tissue‐ and cell‐specific regulation of PPAR‐γ in the lung, where PPAR‐γ is a key lineage‐defining factor for alveolar macrophage differentiation and homeostasis within the GM‐CSF‐dependent niche [36]. In addition, PPAR‐γ function can be modulated independently of its total expression through changes in ligand availability, cofactor recruitment, and post‐translational modifications such as phosphorylation and SUMOylation [37, 38] Therefore, in our model, irradiation may preferentially disrupt PPAR‐γ activity rather than abundance, while differences in tissue type, dominant responding cell populations, radiation dose/fractionation, and sampling time may further contribute to the discrepancies among studies.

Although synthetic PPAR‐γ agonists such as rosiglitazone have demonstrated radioprotective potential in other organs, their application to RILI is restricted by two major hurdles [22, 39, 40]. First, systemic administration is limited by poor pulmonary accumulation and significant off‐target toxicities, including cardiovascular complications and skeletal fragility, which are particularly concerning for patients undergoing radiotherapy [41]. Second, and more importantly, our data reveal a fundamental resistance mechanism: the oxidative microenvironment of RILI compromises the transcriptional activity of PPAR‐γ. Radiation‐induced ROS render PPAR‐γ transcriptionally incompetent through multiple mechanisms, including direct receptor oxidation, depletion of intracellular NAD^+^ pools, and suppression of the transcriptional co‐activator PGC‐1α [42]. Under these conditions, administering conventional ligands encounters a ceiling effect, as the receptor itself is structurally and functionally impaired. Therefore, simply providing a ligand without simultaneously restoring the redox and cofactor environment is ineffective and may require dose escalation, further increasing systemic risks.

To address these physiological and pharmacological challenges, we developed the HANP nanospray with a “triple‐action” mechanism that couples microenvironment remodeling with targeted receptor activation. Initially, the polydopamine shell acts as a broad‐spectrum ROS scavenger, detoxifying the oxidative microenvironment to prevent receptor damage [43, 44]. In parallel, the co‐delivery of NAD^+^ replenishes essential cofactors to reactivate the SIRT1‐AMPK signaling axis [45, 46]. Notably, this axis enhances PPAR‐γ function by promoting its stability and transcriptional competence, effectively “priming” the receptor for subsequent activation [47, 48]. Ultimately, the released ASX serves a dual function, acting not only through its established antioxidant activity but also as a natural ligand that directly binds to and activates the primed PPAR‐γ [49, 50, 51]. Unlike synthetic agonists associated with systemic risks, our strategy utilizes nebulization‐based delivery. This non‐invasive route ensures high pulmonary retention and localized therapeutic action [52, 53, 54], maximizing efficacy while avoiding the cardiovascular and skeletal toxicity common to traditional systemic therapies. Consistent with these observations, our work suggests that sustaining macrophage repolarization depends on restoring mitochondrial quality control—the dynamic balance between clearing damaged organelles (mitophagy) and generating new ones (mitochondrial biogenesis) [55, 56, 57]. Our mechanistic analysis shows that HANP achieves this restoration through a dual‐mechanism strategy. First, regarding clearance, we observed that irradiation induces a significant block in autophagic flux, marked by the pathological co‐accumulation of p62 and LC3‐II. Our findings suggest that HANP potentially relieves this block via a dual‐signaling intervention: delivered NAD^+^ likely fuels SIRT1‐mediated deacetylation to restore the PINK1‐Parkin machinery [58], while released ASX coincides with the suppression of the PI3K‐Akt‐mTOR axis—a negative regulator of autophagy upregulated by radiation [59, 60]. Second, complementing this clearance, HANP promotes mitochondrial renewal through the SIRT1‐AMPK‐PGC‐1α cascade. Significantly, our inhibitor studies indicate a divergent dependency in this process, revealing that while mitophagy is partially driven by NAD^+^/SIRT1 signaling independent of PPAR‐γ, the biogenesis of new mitochondria is strictly dependent upon the PPAR‐γ transcriptional program. Thus, HANP establishes a robust ‘clearance‐renewal’ cycle by coupling mitophagy with PPAR‐γ‐dependent biogenesis. This restores mitochondrial integrity, which metabolically drives the M1–M2 macrophage transition, ultimately resolving chronic inflammation and restoring tissue homeostasis.

Fluorescence imaging showed markedly higher retention of HANP in RILI‐afflicted lungs than in healthy lungs, suggesting preferential accumulation in inflamed pulmonary tissue. This effect is likely multifactorial: radiation‐induced inflammation may increase vascular permeability and impair lymphatic drainage, generating an inflammation‐associated EPR‐like effect that facilitates nanoparticle extravasation and retention; meanwhile, the expanded population of activated macrophages in RILI lungs may further enhance local sequestration through phagocytic uptake. In addition, the melanin‐like HMPDA framework and the abundant phenolic hydroxyl and amino groups on the polydopamine surface may reduce rapid clearance and promote adhesion to inflamed tissue through hydrogen bonding and electrostatic interactions [61, 62]. Localized inhalation delivery may offer several potential translational advantages over systemic radioprotectors such as Amifostine. By depositing HANP directly into the lung, the primary site of radiation‐induced injury, this route may achieve higher local bioavailability while limiting systemic exposure. In contrast, systemic administration of Amifostine is often associated with dose‐limiting adverse effects, including hypotension, nausea, and vomiting. Local pulmonary delivery may therefore reduce off‐target toxicity and improve tolerability. Moreover, inhalational administration may enable more effective modulation of the pulmonary inflammatory microenvironment, including alveolar macrophages, which appear to be central to the therapeutic mechanism of HANP. Importantly, HANP remained effective when administered via different routes in distinct radiation models. Nebulized inhalation was used in the localized thoracic irradiation model to enhance pulmonary deposition and exploit its lung‐targeting advantage, whereas intraperitoneal injection was applied in the whole‐body irradiation model to evaluate systemic radioprotection. The efficacy observed with both delivery routes suggests that HANP has broad therapeutic potential in different radiation injury settings, supporting its future application in both lung‐targeted and systemic radioprotection.

While our study presents a robust, multi‐omics‐guided paradigm for RILI treatment, we acknowledge certain limitations. Primarily, due to the low ex vivo yield of primary alveolar macrophages, we employed peritoneal macrophages as a surrogate model for mechanistic validation. Although these populations differ ontogenetically, the core ROS‐scavenging and PPAR‐γ signaling axes are highly conserved. Importantly, our in vivo scRNA‐seq and immunofluorescence analyses confirmed that the reprogramming observed in vitro is faithfully mirrored within the pulmonary microenvironment. Lastly, regarding target specificity, we relied on the chemical antagonist GW9662 to validate PPAR‐γ dependency. To strictly isolate the contribution of macrophage‐intrinsic PPAR‐γ and rule out potential off‐target effects, future investigations will benefit from employing genetic systems, such as myeloid‐specific *Pparg* knockout mice.

## 4 Conclusions

Although radiation did not markedly alter PPAR‐γ expression, it substantially impaired its transcriptional activity and downstream signaling, identifying functional suppression of PPAR‐γ as a key mechanism underlying RILI progression. To address this, we developed HANP, an inhalable, ROS‐responsive nanospray that enables targeted activation of PPAR‐γ within the pulmonary microenvironment. By scavenging excessive ROS and codelivering NAD^+^ and ASX, HANP restored the SIRT1–PGC‐1α–PPAR‐γ signaling axis, reactivated PPAR‐γ‐dependent transcription, and enhanced PPAR‐γ expression, thereby promoting macrophage reprogramming toward a protective phenotype through improved mitochondrial homeostasis. Ultimately, this targeted nebulization strategy circumvents the systemic toxicity and narrow therapeutic indices of current radioprotectants, offering a robust, non‐invasive, and clinically translatable countermeasure against radiation‐induced tissue damage.

## 5 Experimental Section

### 5.1 Materials

Dopamine hydrochloride, Astaxanthin (ASX, A114383), and the PPAR‐γ selective inhibitor GW9662 (G125880) were acquired from Aladdin Reagents (Shanghai, China). Pluronic F127 (P2443) was obtained from Sigma–Aldrich (St. Louis, MO, USA). 1,3,5‐trimethylbenzene (TMB) was procured from Adamas‐Beta (Shanghai, China). Nicotinamide adenine dinucleotide (NAD^+^, HY‐B0445) and Indocyanine Green (ICG, HY‐D0711) were sourced from MedChemExpress (Princeton, NJ, USA). NH2‐PEG‐COOH was purchased from J&K Scientific (Shenzhen, China). Commercially available assay kits for GSH, SOD, MDA, and BCA protein quantification were supplied by Beyotime Biotechnology (Shanghai, China). The DHE assay kit was acquired from BestBio (Shanghai, China). Primary antibodies against TNF‐α (17590‐1‐AP), IL‐6 (NB600‐1131SS), iNOS (18985‐1‐AP), PPAR‐γ (16643‐1‐AP), PGC‐1α (66369‐1‐Ig), LC3 (14600‐1‐AP), p62 (18420‐1‐AP), PI3K (20584‐1‐AP), AKT (10176‐2‐AP), and fluorophore‐conjugated CD206 (56‐2061‐82) were provided by Proteintech (Rosemont, IL, USA). Antibodies targeting IL‐1β (12242S), p‐AMPK (2535S), mTOR (2972S), and TOMM20 (42406S) were purchased from Cell Signaling Technology (Danvers, MA, USA). PINK1 (DF7742) and Parkin (AF0235) antibodies were sourced from Affinity Biosciences (Cincinnati, OH, USA). Fluorophore‐conjugated flow cytometry antibodies against CD86 (25‐0862‐82), CD11b (11‐0112‐82), F4/80 (12‐4801‐82), and CD45 (17‐0451‐82) were obtained from Thermo Fisher Scientific/Invitrogen (Carlsbad, CA, USA). Anti‐mouse CD16/CD32 (Fc Block) was purchased from BD Pharmingen (San Jose, CA, USA). The nebulizer system was sourced from Yuyan Instruments (Shanghai, China). ELISA kits for TNF‐α (LT‐EK282HS‐96), IL‐6 (LT‐EK206HS‐96), and IL‐1β (YJ696735) were supplied by Langtian (Shanghai, China) and Yuanju Biotechnology (Shanghai, China).

### 5.2 Bioinformatic Analyses

Gene expression microarray datasets of Mus musculus lung tissue were retrieved from the Gene Expression Omnibus (GEO) database (GSE85359, GSE41789, and GSE25295), comprising 28 control and 51 irradiated (IR) samples. The datasets were merged, and batch effects were eliminated using the sva R package (version 3.56.0). Principal component analysis (PCA) was conducted to evaluate the effectiveness of batch‐effect correction. Differentially expressed genes (DEGs) between control and IR groups were identified utilizing the limma package, with P‐values adjusted for multiple testing via the Benjamini–Hochberg false discovery rate (FDR) correction (FDR < 0.05).

ScRNA‐seq data (GSE206426), including 2 control and 2 IR samples, were additionally downloaded from the GEO database. The preprocessed count matrices were imported to construct a Seurat object for downstream processing. Unsupervised clustering was performed, and cell populations were visualized using t‐distributed stochastic neighbor embedding (t‐SNE). Cell type annotation was executed utilizing the SingleR package. Expression profiles of representative marker genes and PPAR‐γ across distinct clusters were visualized using violin plots and t‐SNE feature plots. For quantitative comparison, PPAR‐γ expression levels within the macrophage clusters were statistically assessed using the Wilcoxon rank‐sum test.

### 5.3 Co‐Immunoprecipitation

Cell samples were lysed in IP lysis buffer containing protease inhibitors and deacetylase inhibitors. After centrifugation at 12 000 × g for 15 min at 4°C, the supernatants were collected, and protein concentrations were determined prior to immunoprecipitation. Equal amounts of protein from each sample were incubated overnight at 4°C with an anti‐acetyl‐lysine antibody (9441, Cell Signaling Technology), whereas normal rabbit IgG served as the negative control. Protein A/G magnetic beads (EA‐IP‐007 M, Elabscience) were then added to each sample and incubated for another 3 h at 4°C under gentle rotation. The bead‐bound immune complexes were washed five times with IP lysis buffer, eluted, and subsequently analyzed by immunoblotting using the indicated antibodies.

### 5.4 Synthesis of HANP NPs

HMPDA NPs were synthesized via a modified organic/inorganic self‐assembly method. Briefly, Pluronic F127 (2500 mg) and dopamine hydrochloride (750 mg) were dissolved in a mixture of anhydrous ethanol (75 mL) and ultrapure water (75 mL). Following the addition of TMB (1 mL), the mixture was sonicated for 30 min. Aqueous ammonia (4.5 mL) was added dropwise under continuous stirring (800 rpm) at room temperature for 1 h. The resulting HMPDA NPs were harvested by centrifugation at 15 000 × g for 10 min, washed, and lyophilized.

To construct HANP NPs, HMPDA NPs (1 mg/mL in water) were mixed with ASX (2 mg/mL in DMSO) at a 1:1 volume ratio, sonicated, and stirred for 4 h in the dark. The collected HMPDA@ASX NPs were subsequently incubated with NAD^+^ aqueous solution (10 mg/mL) at a volume ratio of 15:13 for 1 h. For PEGylation, the NAD^+^‐loaded NPs were reacted with NH_2_‐PEG‐COOH (2 mg/mL in pH 8.5 Tris buffer) at a 1:1 volume ratio for 8 h, followed by centrifugation to obtain HANP NPs. ICG‐loaded HANP NPs were synthesized analogously by co‐incubating ICG (0.5 mg) during the loading phase.

### 5.5 Characterization of HANP

The morphology of HANP was examined using transmission electron microscopy (TEM; JEM‐1400plus, JEOL). Hydrodynamic diameter and zeta potential were determined via dynamic light scattering (DLS; Zetasizer Nano ZS90, Malvern) at 25°C. Chemical structure and surface composition were analyzed utilizing UV‐vis‐NIR spectroscopy, Fourier‐transform infrared (FTIR) spectroscopy (Thermo Nicolet iS10), and X‐ray photoelectron spectroscopy (XPS; Thermo K‐Alpha). Crystalline structure was assessed by X‐ray diffraction (XRD; Bruker D8 Advance, Cu Kα radiation). Specific surface area and pore size distribution were calculated using the BET and BJH methods based on nitrogen adsorption–desorption isotherms (Micromeritics ASAP 2020). The droplet size distribution of HANP delivered by a microsprayer aerosolizer (Yuyan Instruments, YAN30012) was analyzed by laser diffraction (Spraylink, LINKOPTKIN).

### 5.6 In Vivo Biodistribution

Specific pulmonary accumulation was evaluated using a near‐infrared (NIR) imaging system (Aniview 100 imaging system, Biolight Biotechnology Co). Following nebulization of ICG‐loaded HANP, NIR imaging was performed at 0, 12, 24, and 48 h. Mice were euthanized for ex vivo imaging of major organs. Fluorescence intensities were quantified utilizing Image Studio software (version 5.2).

### 5.7 In Vivo Animal Models and Treatments

For the RILI model, mice were randomly assigned to four groups (*n* = 5): Control, IR, IR + 25 mg/kg HANP, and IR + 50 mg/kg HANP. Under anesthesia (1% pentobarbital sodium, i.p.), mice were subjected to a single dose of 17 Gy thoracic ^6^ ^0^Co γ‐irradiation (0.35 Gy/min) with the rest of the body lead‐shielded. HANP (25 µL) or saline was administered via pulmonary nebulization (Yuyan Instruments) at 2 h and day 5 post‐irradiation. Pulmonary function was longitudinally assessed using whole‐body plethysmography (Buxco, USA), with investigators blinded to group allocation. Gross lung morphology and lung index were evaluated on day 30. Additionally, high‐resolution micro‐CT scanning (Bruker SkyScan 1276) was performed on day 30 to acquire axial images and generate 3D lung reconstructions for quantifying structural changes, including pulmonary consolidation and fibrosis.

For the PPAR‐γ mechanistic intervention, mice were randomized into four groups (*n* = 8): IR, IR + GW9662, IR + HANP (50 mg/kg), and IR + GW9662 + HANP. The PPAR‐γ antagonist GW9662 (1 mg/kg, i.p.) was administered daily for 7 days pre‐irradiation and continued for 30 days post‐irradiation. Irradiation and HANP nebulization protocols were identical to the RILI model described above.

To further evaluate the effect of HANP on the late‐stage progression of radiation‐induced pulmonary fibrosis, the observation period was extended to 16 weeks after irradiation. At week 16, mice were sacrificed and lung tissues were collected for fibrosis assessment. Masson's trichrome staining was performed to evaluate collagen deposition and the degree of tissue fibrosis, and immunohistochemical staining was conducted to examine the expression of fibrosis‐related markers.

For the whole‐body irradiation survival assay, mice (n = 10) were exposed to a lethal dose of 6 Gy whole‐body ^6^ ^0^Co γ‐irradiation. Treatments, including Amifostine (100 mg/kg, i.p.; positive control) or HANP (100 mg/kg, i.p.), were administered immediately post‐irradiation. Survival rates and body weights were monitored daily for 30 days.

### 5.8 Isolation and Identification of Macrophages

Peritoneal macrophages were elicited by intraperitoneal injection of 3% thioglycollate broth (1 mL) into male BALB/c mice for three consecutive days. On day 4, peritoneal exudate cells were harvested by lavage with cold RPMI‐1640 supplemented with 10% FBS. Cells were plated and allowed to adhere for 2–3 h at 37°C, after which non‐adherent cells were removed by washing with PBS. Macrophage purity was verified by flow cytometry using antibodies against CD45, CD11b, and F4/80 (details in Table S1). Only cell populations demonstrating >90% CD45^+^CD11b^+^F4/80^+^ purity were used for downstream experiments.

### 5.9 Flow Cytometric Analysis of Macrophage Polarization

Primary macrophages exposed to 10 Gy irradiation were subsequently treated with HANP (25 or 50 µg/mL) for 24 h. Cells were harvested, blocked with anti‐CD16/CD32 (Fc Block) for 15 min at 4°C to prevent nonspecific binding, and stained with fluorochrome‐conjugated antibodies against CD45 (APC), CD11b (FITC), F4/80 (PE), CD86 (PE‐Cy7), and CD206 (AF700) for 30 min at 4°C in the dark. Detailed antibody information (vendors, clones, catalog numbers, and dilutions) is provided in Table S1. Following washing, data were acquired on a Sony ID7000 spectral flow cytometer. After excluding debris and doublets, macrophages were defined by the CD45^+^CD11b^+^F4/80^+^ gating strategy. The phenotypic polarization was evaluated by quantifying the proportions of CD86^+^ (M1‐like) and CD206^+^ (M2‐like) subsets using FlowJo software (version 10.9.0, BD Biosciences).

### 5.10 Histological, Immunofluorescence, and Biochemical Analyses

Lung structural changes were evaluated via high‐resolution micro‐CT (Bruker SkyScan 1276) on day 30, with 3D reconstructions and ROI quantifications performed in a blinded manner. Apoptosis and in vivo ROS generation in lung sections were assessed using a TUNEL Apoptosis Kit and Dihydroethidium (DHE) staining (10 µm), respectively. For multiplex immunofluorescence, cells or lung sections were probed with primary antibodies against IL‐1β, IL‐6, TNF‐α, PPAR‐γ, CD36, and Arg1, followed by appropriate fluorophore‐conjugated secondary antibodies. Images were acquired via confocal microscopy (Nikon A1R) and quantified using ImageJ software (version 1.53e). Levels of oxidative stress markers (MDA, SOD, GSH) and cytokines (IL‐1β, IL‐6, TNF‐α) in lung homogenates were quantified using commercial assay kits and ELISA kits, respectively, normalized to BCA total protein content.

### 5.11 Immunohistochemistry

Paraffin‐embedded tissue sections were first deparaffinized in xylene and rehydrated in a graded series of ethanol. Antigen retrieval was carried out by heating the sections in citrate buffer (pH 6.0). To quench endogenous peroxidase activity, the sections were treated with 3% hydrogen peroxide for 10 min, followed by blocking with 5% goat serum for 1 h at room temperature to reduce nonspecific staining. The sections were then incubated overnight at 4°C with primary antibodies against α‐SMA, TGF‐β, and Collagen I. After rinsing with PBS, the sections were exposed to HRP‐conjugated secondary antibodies for 1 h at room temperature. Immunostaining was visualized with 3,3′‐diaminobenzidine, and the nuclei were counterstained with hematoxylin. Finally, all sections were dehydrated, coverslipped, and examined under a light microscope.

### 5.12 Western Blot Analysis

Protein lysates were extracted using RIPA buffer containing protease and phosphatase inhibitors. Equal amounts of protein (30 µg), quantified via BCA assay, were resolved by SDS‐PAGE and transferred to PVDF membranes. Membranes were probed overnight at 4°C with specific primary antibodies against iNOS, IL‐1β, IL‐6, TNF‐α, PGC‐1α, PPAR‐γ, LC3, p62, PI3K, AKT, mTOR, p‐AMPK, TOMM20, Parkin, PINK1, α‐SMA, TGF‐β, Smad3, CD36, Arg1 and β‐actin. Detailed antibody information (vendors, catalog numbers, clones, and dilutions) is listed in Table S2. Protein bands were visualized using ECL reagents on an imaging system (Bio‐Rad) and quantified using Image Lab software (version 4.0).

### 5.13 Transcriptomic Sequencing and Analysis

Total RNA was extracted from lung tissues (day 30) using TRIzol reagent. Following quality control (Agilent 2100, RIN > 7.0), poly(A)‐enriched cDNA library construction and paired‐end sequencing (150 bp) were performed on an Illumina NovaSeq 6000 platform by Bioprofile Technologies (Shanghai, China). For bioinformatic analysis, raw reads were filtered using Fastp and mapped to the Mus musculus reference genome (GRCm38) using HISAT2. DEGs were identified using DESeq2, with significance criteria set at |log_2_FC| > 1 and adjusted *p* < 0.05. Functional enrichment analyses (GO and KEGG) were conducted utilizing the clusterProfiler package.

### 5.14 Statistical Analysis

Statistical analyses were executed utilizing GraphPad Prism (version 9.0). Data are presented as mean ± s.d. Data normality and variance homogeneity were evaluated using the Shapiro–Wilk and Brown–Forsythe tests, respectively. Comparisons between two groups were analyzed via unpaired two‐tailed Student's *t*‐test, whereas multiple comparisons were assessed using one‐way ANOVA followed by Tukey's post hoc test. *p* < 0.05 was considered statistically significant.

## Author Contributions

**Mingquan Gao**: conceptualization, methodology, data curation, investigation, formal analysis, funding acquisition, writing – original draft. **Xudong Yu**: methodology, software, data curation, investigation, validation, formal analysis, visualization. **Ziqian Shang**: methodology, data curation, investigation, validation, formal analysis. **Mengyao Tan**: methodology, software, formal analysis, visualization. **Xie Huang**: data curation, investigation, validation. **Zaizhi Du**: investigation, validation, project administration. **Xiaojiao Wang**: data curation, investigation, formal analysis. **Xinrui Yang**: software, data curation. **Ximei Luo**: methodology, software, validation, formal analysis, resources. **Weidong Wang**: conceptualization, supervision, project administration, writing – review & editing. **Rong Li**: conceptualization, supervision, project administration, resources, writing – review & editing. **Shenglin Luo**: conceptualization, supervision, funding acquisition, project administration, resources, writing – original draft, writing – review & editing.

## Funding

This study was supported by the National Natural Science Foundation of China (82404198 and 82173457).

## Conflicts of Interest

The authors declare no conflicts of interest.

## Supporting information




**Supporting File**: advs76447‐sup‐0001‐SuppMat.docx.

## Data Availability

The data that support the findings of this study are available on request from the corresponding author. The data are not publicly available due to privacy or ethical restrictions.
