# Supplementary material for: Inhalable ROS‐Responsive Nanospray Activates PPAR‐γ to Restore Macrophage Mitochondrial Homeostasis and Attenuate Radiation‐Induced Lung Injury
Source: Adv Sci (Weinh). 2026 Jul 8;13(54):e76447. doi: 10.1002/advs.76447 (PMC13344066; doi:10.1002/advs.76447)
Supplement: Supplementary file 1 [file ADVS-13-e76447-s001.docx]

**Supplementary Material**

**Inhalable ROS-responsive nanospray activates PPAR-γ to restore macrophage mitochondrial homeostasis and attenuate radiation-induced lung injury**

Mingquan Gao^a†^**,** Xudong Yu^a†^**,** Ziqian Shang^a^**,** Mengyao Tan^b^**,** Xie Huang^a^**,** Zaizhi Du^a^**,** Xiaojiao Wang^a^**,** Xinrui Yang^a^**,** Ximei Luo^b^**,** Weidong Wang^c*^**,** Rong Li^a*^**,** Shenglin Luo^a*^

^a^Institute of Combined Injury, State Key Laboratory of Trauma and Chemical Poisoning, Chongqing Engineering Research Center for Nanomedicine, Department of Military Preventive Medicine, Third Military Medical University (Army Medical University), Chongqing 400038, China.

^b^Institute of Fundamental and Frontier Sciences, University of Electronic Science and Technology of China, Chengdu 610054, China.

^c^Department of Radiation Oncology, Radiation Oncology Key Laboratory of Sichuan Province, Sichuan Clinical Research Center for Cancer, Sichuan Cancer Hospital & Institute, Affiliated Cancer Hospital of University of Electronic Science and Technology of China, Chengdu 610000, China.

^†^These authors contributed equally to this work.

**^*^Correspondence:** Shenglin Luo (luosl@tmmu.edu.cn), Rong Li (lrong361@126.com), Weidong Wang (wwdwyl@uestc.edu.cn)

Contents

[Figure S1.	3](#_Toc231420581)

[Figure S2.	4](#_Toc231420582)

[Figure S3.	5](#_Toc231420583)

[Figure S4.	6](#_Toc231420584)

[Figure S5.	7](#_Toc231420585)

[Figure S6.	8](#_Toc231420586)

[Figure S7.	9](#_Toc231420587)

[Figure S8.	10](#_Toc231420588)

[Figure S9.	11](#_Toc231420589)

[Figure S10.	12](#_Toc231420590)

[Figure S11.	13](#_Toc231420591)

[Figure S12.	14](#_Toc231420592)

[Figure S13.	15](#_Toc231420593)

[Figure S14.	16](#_Toc231420594)

[Figure S15.	17](#_Toc231420595)

[Figure S16.	18](#_Toc231420596)

[Figure S17.	19](#_Toc231420597)

[Figure S18.	20](#_Toc231420598)

[Figure S19.	21](#_Toc231420599)

[Figure S20.	22](#_Toc231420600)

[Figure S21.	23](#_Toc231420601)

[Figure S22.	24](#_Toc231420602)

[Figure S23.	25](#_Toc231420603)

[Figure S24.	27](#_Toc231420604)

[Figure S25.	28](#_Toc231420605)

[Figure S26.	29](#_Toc231420606)

[Figure S27.	30](#_Toc231420607)

[Figure S28.	31](#_Toc231420608)

[Figure S29.	33](#_Toc231420609)

[Figure S30.	34](#_Toc231420610)

[Figure S31.	35](#_Toc231420611)

[Figure S32.	36](#_Toc231420612)

[Figure S33.	38](#_Toc231420613)

[Figure S34.	39](#_Toc231420614)

[Table S1.	40](#_Toc231420615)

[Table S2.	41](#_Toc231420616)

**Figure S1. Multi-omics profiling identifies stable PPAR-γ expression patterns in RILI.**

**a** PCA of integrated transcriptomic datasets. **b** Volcano plot of DEGs between control and IR groups. **c** PPAR-γ mRNA levels in bulk tissue. **d** Single-cell RNA-seq analysis (GSE206426) showing cell-type composition. **e** t-SNE visualization of PPAR-γ expression.

**Figure S2. Ionizing radiation promotes PPAR-γ acetylation but does not affect total PPAR-γ expression in macrophages. a** Representative immunofluorescence images of PPAR‑γ expression/localization in MH-S cells after irradiation. **b-c** Representative western blot images and quantitative analysis of total PPAR‑γ, acetylated PPAR‑γ in MH-S cells after irradiation. Data are presented as mean ± s.d. Statistical analysis was performed using independent samples t-test. **P* < 0.05, ***P* < 0.01.

**Figure S3. TEM images of HANP preparation under different stirring speeds and times. a–e** were obtained using low-speed stirring for 30 min, 1 h, 2 h, 4 h, and 6 h, respectively. **f** was obtained using high-speed stirring for 1 h.

**Figure S4. Nitrogen adsorption-desorption isotherm of HMPDA.** The inset shows the pore size distribution derived from the adsorption branch.

**Figure S5.** **TEM images of HANP under different pH and H₂O₂ conditions.** **a** pH 7.4. **b** pH 7.4 + H₂O₂. **c** pH 6.8.

**Figure S6. Stability evaluation of HANP under different storage times and pH values. a** Changes in hydrodynamic diameter and polydispersity index of HANP during storage at 37 °C over 1–5 days. **b** Hydrodynamic diameter and PDI of HANP after storage for 24 h at different pH values (5.5, 6.5, and 7.4).

**Figure S7. Aerosol characterization of nebulized HANP**. **a** Characteristic droplet diameters of nebulized HANP, including D10, D50, and D90, summarizing the particle size distribution profile. **b** Volume-based droplet size distribution of HANP after nebulization, measured by laser diffraction.

**Figure S8. Mucus-penetrating behavior of HANP. a** Representative images of HANP penetration through the mucus layer. **b** Quantitative analysis of HANP transport across a mucus-coated Transwell system (n = 3).

**Figure S9. Lung distribution of HANP after intratracheal nebulization.** Representative fluorescence image of frozen lung sections after administration of ICG-labeled HANP. Red, HANP-ICG; blue, DAPI. Scale bar: 100 μm.

**Figure S10. Pulmonary function parameters measured by whole-body plethysmography at the first week post-irradiation under different treatment conditions (n = 4)**, including **a** respiratory frequency, **b** tidal volume, **c** minute volume, **d** enhanced pause (Penh), **e** pause, **f** peak inspiratory flow, **g** peak expiratory flow, and **h** forced expiratory flow at 50% of tidal volume (FEF_50_). Data are presented as mean ± s.d. Statistical analysis was performed using one-way ANOVA with Tukey’s post hoc test. **P* < 0.05.

**Figure S11. Pulmonary function parameters measured by whole-body plethysmography at the second week post-irradiation under different treatment conditions (n = 4)**, including **a** respiratory frequency, **b** tidal volume, **c** minute volume, **d** enhanced pause (Penh), **e** pause, **f** peak inspiratory flow, **g** peak expiratory flow, and **h** forced expiratory flow at 50% of tidal volume (FEF_50_). Data are presented as mean ± s.d. Statistical analysis was performed using one-way ANOVA with Tukey’s post hoc test. ***P* < 0.01, ****P* < 0.001.

**Figure S12.** **Pulmonary function parameters measured by whole-body plethysmography at the third week post-irradiation under different treatment conditions (n = 4)**, including **a** respiratory frequency, **b** tidal volume, **c** minute volume, **d** enhanced pause (Penh), **e** pause, **f** peak inspiratory flow, **g** peak expiratory flow, and **h** forced expiratory flow at 50% of tidal volume (FEF_50_). Data are presented as mean ± s.d. Statistical analysis was performed using one-way ANOVA with Tukey’s post hoc test. **P* < 0.05, ***P* < 0.01, ****P* < 0.001.

**Figure S13. Pulmonary function parameters measured by whole-body plethysmography at the fourth week post-irradiation under different treatment conditions (n = 4)**, including **a** minute volume, **b** pause, **c** peak inspiratory flow, and **d** peak expiratory flow. Data are presented as mean ± s.d. Statistical analysis was performed using one-way ANOVA with Tukey’s post hoc test. **P* < 0.05, ***P* < 0.01, ****P* < 0.001.

**Figure S14. Gating strategy for the identification of macrophages in mouse lung tissue by flow cytometry.** Lymphocytes were first gated based on FSC/SSC profiles, followed by selection of single cells. CD45⁺ leukocytes were then identified, and CD11b⁺F4/80⁺ double-positive cells were subsequently gated as lung macrophages.

**Figure S15. Gating strategy for the identification of macrophages from isolated cells by flow cytometry.** Lymphocytes were first gated based on forward and side scatter (FSC/SSC) characteristics, followed by selection of single cells. CD45⁺ immune cells were then identified, and CD11b⁺F4/80⁺ double-positive cells were gated as macrophages. Subsequent gates were applied for downstream subpopulation analysis.

**Figure S16. Live/dead staining to assess HANP cytotoxicity on isolated macrophages.** Calcein AM (green) stains live cells, while PI (red) marks dead cells. Scale bars: 100 µm.

**Figure S17. Live/dead staining of isolated macrophages after 10 Gy irradiation with or without HANP treatment (25 or 50 μg/mL).** Calcein AM (green) stains live cells, while PI (red) marks dead cells. Scale bar: 20 μm.

**Figure S18. HANP reduces radiation-induced ROS production in isolated macrophages (n = 3). a** DCFH-DA fluorescence staining of macrophages after 10 Gy irradiation with or without HANP treatment (25 or 50 μg/mL). **b** Quantification of ROS fluorescence intensity. Scale bar: 20 μm. Data are presented as mean ± s.d. Statistical analysis was performed using one-way ANOVA with Tukey’s post hoc test. **P* < 0.05, ***P* < 0.01, ****P* < 0.001.

**Figure S19.** **HANP attenuates radiation-induced increase in intracellular calcium levels in isolated macrophages (n = 5). a** Fluo-4 AM calcium fluorescence staining of isolated macrophages after 10 Gy irradiation with or without HANP treatment (25 or 50 μg/mL). Green fluorescence indicating increased calcium levels. **b** Quantification of Ca²⁺ fluorescence intensity. Scale bar: 20 μm. Data are presented as mean ± s.d. Statistical analysis was performed using one-way ANOVA with Tukey’s post hoc test. **P* < 0.05, ***P* < 0.01, ****P* < 0.001.

**Figure S20. HANP maintains mitochondrial membrane potential in irradiated macrophages (n = 3). a** JC-1 fluorescence staining of mitochondrial membrane potential in macrophages after 10 Gy irradiation with or without HANP treatment (25 or 50 μg/mL). JC-1 aggregates (red) indicating healthy mitochondria and JC-1 monomers (green) indicating depolarized mitochondria. Scale bar: 20 μm. **b** Quantification of the red/green fluorescence intensity ratio. Data are presented as mean ± s.d. Statistical analysis was performed using one-way ANOVA with Tukey’s post hoc test. **P* < 0.05, ***P* < 0.01, ****P* < 0.001.

**Figure S21. HANP alleviates DNA damage in irradiated macrophages (n = 3). a** Immunofluorescence staining of γ-H2AX in macrophages following 10 Gy irradiation, with or without HANP treatment (25 or 50 μg/mL). DAPI (blue) stains the nuclei, and γ-H2AX foci (green) indicate DNA double-strand breaks. Scale bar: 20 μm. **b** Quantification of γ-H2AX foci per nucleus under each condition. Data are presented as mean ± s.d. Statistical significance was determined by one-way ANOVA followed by Tukey’s post hoc test. **P* < 0.05, ***P* < 0.01, ****P* < 0.001.

**Figure S22. HANP inhibits the PI3K/AKT/mTOR signaling pathway in irradiated macrophages (n = 3).** **a** Representative western blot images showing the expression levels of PI3K, AKT, and mTOR in macrophages from control, irradiated (IR), and IR treated with 25 µg/mL and 50 µg/mL HANP. **b-d** Quantification of protein expression levels for PI3K, AKT, and mTOR. Data are presented as mean ± s.d. Statistical significance was determined by one-way ANOVA followed by Tukey’s post hoc test. **P* < 0.05, ***P* < 0.01, ****P* < 0.001.

**Figure S23. Validation of HANP effects on macrophage polarization and mitochondrial regulatory pathways in MH-S cells. a** Representative flow cytometric contour plots of CD86/CD206 staining in MH-S cells. **b** Quantification of CD86-positive and CD206-positive cell populations (n = 3). **c** Representative western blot images of PPAR-γ, PGC-1α, SIRT1, PINK1, Parkin, p62, TFAM, and TOMM20. **d-f** Quantitative analysis of PPAR-γ, PGC-1α, SIRT1, PINK1, Parkin, p62, TFAM, and TOMM20 expression (n = 3). Data are presented as mean ± s.d. Statistical analysis was performed using one-way ANOVA with Tukey’s post hoc test. **P* < 0.05, ***P* < 0.01, ****P* < 0.001.

**Figure S24. HANP restores PPAR‑γ expression, nuclear localization, and downstream signaling in irradiated MH-S cells. a** Representative immunofluorescence images of PPAR‑γ in MH-S cells after irradiation. Scale bar: 2.5 μm. **b** Quantitative analysis of PPAR‑γ transcription factor activity in MH-S cells after irradiation (n = 3). **c-e** Representative western blot images and quantitative analysis of CD36 and Arg1 expression in MH-S cells after irradiation (n = 3). Data are presented as mean ± s.d. Statistical analysis was performed using one-way ANOVA with Tukey’s post hoc test. **P* < 0.05, ***P* < 0.01, ****P* < 0.001.

**Figure S25.** **PPAR-γ inhibition attenuates the HANP-induced improvement of lung function in irradiated mice** **(n = 4). a** Minute volume (mL/L·min), **b**: Pause, **c**: Peak inspiratory flow (mL/L·sec), **d**: Peak expiratory flow (mL/L·sec). Groups include (1) IR, (2) IR + GW9662, (3) IR + HANP, and (4) IR + GW9662 + HANP. Data are presented as mean ± s.d. Statistical significance was determined by one-way ANOVA followed by Tukey’s post hoc test. **P* < 0.05, ***P* < 0.01, ****P* < 0.001.

**Figure S26.** **Effect of PPAR-γ inhibition on HANP-mediated inflammatory regulation in irradiated macrophages (n = 3).** **a-b** Quantification of protein expression levels for iNOS, IL-1β, IL-6, and TNF-α. Data are presented as mean ± s.d. Statistical significance was determined by one-way ANOVA followed by Tukey’s post hoc test. **P* < 0.05, ***P* < 0.01, ****P* < 0.001.

**Figure S27.** **Effect of PPAR-γ inhibition on HANP-mediated mitochondrial homeostasis in irradiated macrophages** **(n = 3).** **a-b** Quantification of protein expression levels for PINK1, Parkin, LC3, p62, TFAM, and TOMM20. Data are presented as mean ± s.d. Statistical significance was determined by one-way ANOVA followed by Tukey’s post hoc test. **P* < 0.05, ***P* < 0.01, ****P* < 0.001.

**Figure S28.** **Effect of PPAR-γ inhibition on HANP-mediated modulation of the PI3K/AKT/mTOR signaling pathway in irradiated macrophages (n = 3).** **a** Representative western blot images showing the expression levels of PI3K, AKT, and mTOR in macrophages from control, irradiated (IR), and IR + HANP, IR + GW9662 + HANP groups. **b-d** Quantification of protein expression levels for PI3K, AKT, and mTOR. Groups include (1) Control, (2) IR, (3) IR + HANP, and (4) IR + GW9662 + HANP. Data are presented as mean ± s.d. Statistical significance was determined by one-way ANOVA followed by Tukey’s post hoc test. **P* < 0.05, ***P* < 0.01, ****P* < 0.001.

**Figure S29. HANP attenuates late-stage radiation-induced pulmonary fibrosis. a** Representative images of Masson’s trichrome staining and immunohistochemical staining for α-SMA, TGF-β, and Collagen I in lung sections from the indicated groups at 16 weeks after irradiation, Scale bar: 50 μm. **b** Western blot analysis of fibrosis-related proteins, including TGF-β, Smad3, and α-SMA, in lung tissues collected at 16 weeks post-irradiation. **c-e** Quantitative analysis of relative protein expression levels in the indicated groups (n = 3). Data are presented as mean ± s.d. Statistical analysis was performed using one-way ANOVA with Tukey’s post hoc test. **P* < 0.05, ***P* < 0.01, ****P* < 0.001.

**Figure S30.** **The radioprotective effects of HANP in TBI model.** **a** Changes in body weight of mice over 26 days following 6.5 Gy TBI under different treatment conditions (n = 8). Body weight data are presented as mean ± s.d. **b** Survival probability of mice post-irradiation across the same treatment groups (n = 8). Survival differences were analyzed using the log-rank test.

**Figure S31.** **Hematopoietic protective effect of HANP in a TBI model.** Hematological profiles of mice following 6.5 Gy TBI under different treatment conditions (n = 8), including **a** platelet (PLT), **b** red blood cell (RBC), **c** white blood cell (WBC), and **d** neutrophil (NE) counts measured at various time points post-irradiation. Data are presented as mean ± s.d.

**Figure S32. Biological safety evaluation of HANP**. **a** Lung coefficient of healthy mice on day 28 after administration of HANP at 25 or 50 mg/kg (n = 3). Representative images of excised lungs from each group are shown above the bar graph. **b** Body weight changes of healthy mice monitored over 28 days post-treatment (n = 6). Hematological parameters including **c** WBC, **d** RBC, and **e** PLT, measured on days 0, 7, 14, and 21 (n = 3). Data are presented as mean ± s.d. Statistical significance was determined by one-way ANOVA followed by Tukey’s post hoc test.

**Figure S33. Histopathological evaluation of HANP tissue safety**. Hematoxylin and eosin (H&E) staining of major organs (heart, liver, spleen, lung, and kidney) collected from healthy mice on day 28 after administration of HANP at 25 or 50 mg/kg. Scale bar:100 μm.

**Figure S34. Evaluation of the blood compatibility of HANP (n = 4).** **a** Representative images of hemolysis assays evaluating the hemocompatibility of HANP at various concentrations (6.25–100 μg/mL). H₂O and PBS were used as positive and negative controls, respectively. **b** Quantification of hemolysis percentage after treatment with HANP. Data are presented as mean ± s.d.

**Table S1. Fluorochrome-conjugated antibodies used for flow cytometry**

| **Antibodies** | **Fluorophore** | **Host Species** | **Clone ID** | **Manufacturer** | **Catalog Number** | **Dilution** |
| --- | --- | --- | --- | --- | --- | --- |
| CD45 | APC | Rat / IgG2b, kappa | 30-F11 | Invitrogen | 17-0451-82 | 0.125 µg/test |
| F4/80 | PE | Rat / IgG2a, kappa | BM8 | Invitrogen | 12-4801-82 | 0.25 µg/test |
| CD11b | FITC | Rat / IgG2b, kappa | M1/70 | Invitrogen | 11-0112-82 | 0.5 µg/test |
| CD86 | PE-Cyanine7 | Rat / IgG2a, kappa | GL1 | Invitrogen | 25-0862-82 | 0.25 µg/test |
| CD206 | Alexa Fluor™ 700 | Rat / IgG2b, kappa | MR6F3 | Invitrogen | 56-2061-82 | 0.25 µg/test |

**Table S2. Primary antibodies used for western blot analysis**

| **Antibodies** | **Host Species** | **Manufacturer** | **Catalog Number** | **Dilution** |
| --- | --- | --- | --- | --- |
| IL-6 | Rabbit | NOVUS | NB600-1131SS | 1:1000 |
| IL-1β | Mouse | Cell Signaling Technology | 12242S | 1:1000 |
| PGC-1α | Mouse | Proteintech | 66369-1-Ig | 1:5000 |
| LC3 | Rabbit | Proteintech | 14600-1-AP | 1:2000 |
| TOMM20 | Rabbit | Cell Signaling Technology | 42406S | 1:1000 |
| TNF-α | Rabbit | Proteintech | 17590-1-AP | 1:1000 |
| p-AMPK | Rabbit | Cell Signaling Technology | 2535S | 1:1000 |
| PINK1 | Rabbit | Affinity | DF7742 | 1:1000 |
| Parkin | Rabbit | Affinity | AF0235 | 1:500 |
| p62 | Rabbit | Proteintech | 18420-1-AP | 1:5000 |
| TFAM | Rabbit | Proteintech | 22586-1-AP | 1:5000 |
| iNOS | Rabbit | Proteintech | 18985-1-AP | 1:2000 |
| PPAR-γ | Rabbit | Proteintech | 16643-1-AP | 1:2000 |
| mTOR | Rabbit | Cell Signaling Technology | 2972S | 1:1000 |
| PI3K | Rabbit | Proteintech | 20584-1-AP | 1:500 |
| AKT | Rabbit | Proteintech | 10176-2-AP | 1:2000 |
| α-SMA | Mouse | ChangYi biological | AF10987 | 1:1000 |
| Smad3 | Rabbit | ChangYi biological | AF14583 | 1:1000 |
| TGF-β | Rabbit | Zenbio | 346599 | 1:1000 |
| CD36 | Rabbit | Zenbio | R381350 | 1:1000 |
| Arg1 | Rabbit | Zenbio | R380744 | 1:1000 |
